# Hepatocyte SLC31A1-dependent copper accumulation contributes to hepatic glucose and lipid metabolic disturbances through AMPKα1 inactivation

**DOI:** 10.1016/j.bbrep.2026.102787

**Published:** 2026-09-11

**Authors:** Yin-Qin Cheng, Min Wei, Le Yang, You-Yi Zhuang, Qing-Bo Lu

**Affiliations:** a Department of Endocrinology, Nantong Second People's Hospital, Nantong, Jiangsu, China; b Department of Medicine, Wuxi Medical School, Jiangnan University, Wuxi, Jiangsu, China

**Keywords:** Insulin resistance, Hepatic steatosis, SLC31A1, Copper homeostasis, AMPK, Type 2 diabetes

## Abstract

Hepatic insulin resistance and fat accumulation are common features of metabolic syndrome, obesity and type 2 diabetes (T2D). However, whether the copper importer SLC31A1 has a role in diabetes-associated hepatic metabolic disorders remains unknown. Thus, we studied the role of hepatocyte SLC31A1 in HFD/STZ-induced T2D mice and in glucosamine (GlcN)- or oleic acid-treated hepatocytes. Glucose homeostasis and insulin resistance were analysed by determination of blood glucose levels fasting and insulin levels, HOMA insulin resistance (HOMA-IR), intraperitoneal glucose tolerance test (IPGTT) and insulin tolerance test (ITT). Hepatic SLC31A1 expression and accumulation of copper were enhanced in T2D mice with altered expression of cuproptosis-associated genes. Hepatocyte-specific SLC31A1 knockdown improved glucose tolerance and insulin resistance, decrease hepatic gluconeogenesis, restore hepatic glycogen accumulation and attenuate hepatic steatosis, oxidative stress, inflammation and liver injury in mice. Consistently, SLC31A1 knockdown decreased GlcN-mediated gluconeogenesis and glycogen depletion and reduced oleic acid-mediated accumulation of hepatic triglyceride in HepG2 cells. Mechanistically, SLC31A1 knockdown exclusively restored AMPKα1 phosphorylation in metabolically stressed hepatocytes, and genetic AMPKα1 depletion partly rescued the effect of SLC31A1 knockdown on glucose production and TG accumulation. Pharmacological inhibition of AMPK partially abolished the effect of SLC31A1 downregulation in T2D mice. Collectively, hepatocyte SLC31A1 upregulation caused hepatic copper accumulation and contributed to hepatic metabolic dysfunction, at least in part, through inhibition of AMPKα1 signaling.

## Highlights

• Hepatic SLC31A1 and copper levels are upregulated in diabetic livers.
• SLC31A1 knockdown ameliorates insulin resistance and glucose-lipid dysmetabolism.
• SLC31A1 ablation attenuates oxidative stress and hepatic inflammation.
• AMPKα1, but not AMPKα2, mediates the effects of SLC31A1 knockdown on diabetes.

## 1 Introduction

Diabetes is a metabolic disorder that is typically characterized by chronic hyperglycemia resulting from impaired insulin secretion and function [1]. Development and progression of type 2 diabetes (T2D) are closely related to insulin resistance, which reduces insulin sensitivity and disrupts insulin signaling [2]. Diabetes was proved to induce insulin resistance through multifactorial pathwayss, such as genetic background, hyperinsulinemia, high circulating non-esterified fatty acids (NEFA) and glycerol, mitochondrial dysfunction, oxidative and endoplasmic reticulum stress, inflammation, and gut microbiota alteration and so on [[3], [4], [5]]. Despite this knowledge, effective therapies of diabetes are still lacking, largely owing to unclear molecular mechanisms. Finding the potential molecular target or pathway could lead us towards ways to prevent diabetes.

Like other organs involved in glucose homeostasis, the liver is especially responsive to insulin. Hepatic insulin resistance results in glucose excess, lipid accumulation and glucose imbalance [6,7]. As the first organ to which the pancreatic insulin acts, the liver controls glucose balance by modulating gluconeogenesis, glycogenesis and glycogenolysis [8]. For example, during fasting, hormones increase gluconeogenesis by the main gluconeogenesis-related enzymes, such as phosphoenolpyruvate carboxykinase (PEPCK) and glucose-6-phosphatase (G6Pase) [9,10]. Increasing gluconeogenesis-related enzymes inhibits the flux of glycogenesis to accumulate glucose and eventually leads to hyperglycaemia. Dysregulated hepatic lipid metabolism induces liver steatosis, thereby aggravating hepatic insulin resistance and hepatic inflammatory reaction [11]. Control of lipid degradation (fatty acid oxidation, FAO) and lipogenesis is vital for the accurate maintenance of hepatic metabolic homeostasis [12,13]. Enhanced lipogenesis and defect of FAO result in the accumulation of lipid in the liver to induce steatosis [14,15]. Despite our incomplete understanding of the molecular mechanisms of hepatic FAO and lipogenesis, no medication exists to prevent or treat hepatic steatosis. As liver dysfunction is a dominant cause of diabetes pathogenesis, improvement of hepatic insulin sensitivity or steatosis could be a potentially effective therapy of diabetes.

Liver is a main organ for global metabolic and copper homeostasis [16,17]. Recent studies suggest that dysfunction of copper metabolism, due to either environmental or genetic reasons, plays a role in diabetes [18]. Emerging studies suggest a role for copper dyshomeostasis and cuproptosis pathways in diabetic susceptibility and progression [[18], [19], [20]]. SLC31A1, also called copper transporter 1 (CTR1), is a high-affinity copper importer involved in the cellular import of copper [21]. As copper is an essential cofactor for cellular respiration, mitochondrial enzymes and many other metabolic enzymes, changes in the expression or activity of SLC31A1 would affect cellular copper supply, mitochondrial function, redox homeostasis and metabolic-related energy-sensitive pathways [22]. However, whether hepatocyte SLC31A1 is responsible for diabetes-related hepatic glucose and lipid metabolic dysfunction is unclear.

Curopptosis offers a possible mechanism linking copper accumulation to mitochondrial metabolic injury [23,24]. This distinct copper-dependent mode of cell death associated with copper binding to lipoylated tricarboxylic-acid-cycle (TCA) cycle proteins, mainly DLAT, leading to abnormal protein aggregation, loss of iron–sulfur-cluster proteins, mitochondrial proteotoxic stress and cell death [25]. Cells with active mitochondrial respiration and abundant lipoylated TCA cycle proteins are particularly susceptible to this process. These findings point to a role for excessive SLC31A1-dependent copper accumulation in disrupting the metabolic homeostasis of hepatocytes, through mitochondrial and cuproptosis-associated mechanisms. Increased SLC31A1 expression or copper accumulation alone is not sufficient for cuproptosis and the contribution of this cell death mechanism to liver dysfunction in diabetes is not yet clear. Therefore, on this basis, we hypothesized that enhanced hepatic SLC31A1 expression and in turn copper homeostasis disruption could be involved in diabetes-related perturbation of glucose and lipid metabolisms.

## 2 Materials and methods

### 2.1 Reagent and chemicals

Streptozotocin (STZ), glucosamine (GlcN), insulin, and glucose were purchased from Sigma-Aldrich (St. Louis, MO, USA). Total cholesterol (TC) and triglyceride (TG) assay kits were purchased from Mindrasy (Shenzhen, China). Dulbecco's Modified Eagle's Medium (DMEM), fetal bovine serum (FBS), trypsin-EDTA, and penicillin and streptomycin solution were purchased from HyClone (South Logan, UT, USA). The high-fat diet (D12492, 60 kcal% fat) was procured from Research Diets (New Brunswick, NJ, USA). Copper colorimetric assay kit and cuprous fluorometric assay kit were obtained from Elabscience (Wuhan, China). Hematoxylin and eosin (H&E) staining kit, Oil O Red staining kit, G6pase activity kit, and PEPCK activity kits, periodic acid-schiff (PAS) kit, nonestesterified fatty acid (NEFA) assay kit, and alanine aminotransferase (ALT) assay kit, aspartate aminotransferase (AST) assay kit, glucose assay kit, insulin assay kit, and glycogen assay kit were obtained from Jiancheng (Nanjing, China). Enzyme-linked immunosorbent assay (ELISA) kit for IL-1β (interleukin-1β), IL-6 (interleukin-6) and TNF-α (tumour necrosis factorα) was provided by BOSTER Biological Technology (Wuhan, China). Measurement kits for SOD (superoxide dismutase), GPx (glutathione peroxidase) and MDA (malondialdehyde) were purchased from Solarbio LIFE SCIENCES (Beijing, China). Primary antibodies for the following proteins were provided by Cell Signaling Technology (Danvers, MA, USA): AMPKα1 (#2795), phospho-AMPKα1 (Ser485) (#4184), AMPKα2 (#2757), F4/80 (#30325), Ly6g (#88876), Nrf2 (#12721) and β-actin (#4967). SLC31A1 polyclonal antibody was purchased from Proteintech (Wuhan, China). Primary antibody to phospho-AMPKα2 (Ser345) (ab129081) was purchased from Abcam (Cambridge, UK). Control shRNA (sc-108060) and SLC31A1 shRNA (sc-105249-SH) were purchased from Santa Cruz (Santa Cruz, CA, USA). Control siRNA (siN0000001-4-10), AMPKα1 siRNA (siG000005571A-1-5), AMPKα2 siRNA (siG000051422B-1-5) were purchased from RIBOBIO (Guangzhou, China). Lipofectamine transfection reagent, RIPA lysis buffer, and the BCA protein quantification kit were obtained from by Thermo Fisher Scientific (Waltham, MA, USA). LY294002, MK2206, and Dorsomorphin were bought from Selleck Chemicals (Houston, TX, USA).

### 2.2 Animals

Six-week-old male C57BL/6J mice (20–22g) were purchased from SPF Biotechnology Co. Ltd (Shanghai, China). Sixty mice were housed together in housing cages under a humidity and temperature controlled environment in a 12-h light and dark cycle and with free access to standard chow and clean water. Twelve mice were used to detect the expression of cuproptosis-related genes in the liver. Twenty-four mice were used to analyse the effect of SLC31A1 knockdown on diabetes. Twenty-four mice were employed to investigate whether AMPK1 inhibition negated the beneficial effect of SLC31A1 knockdown on diabetes. Sizes of the groups were determined based on previous studies using the HFD/STZ model and n = 6 mice for each group [[26], [27], [28]]. The animal status, behaviour and activity were monitored twice a day during the entire experimental period, once in the morning and once in the afternoon. All animal procedures conformed to the institutional requirements, and our research staff received specific training on animal manipulation, operating procedures of surgery, care during the post-surgery period. All animal experiments performed in Jiangnan University. No animals were excluded on group allocation after assignment and no animals were spontaneously dead from the study. This study strictly adhered to the guidelines of ARRIVE guidelines. and this experiment complied with the regulation of Care and Use of Laboratory Animals published by the US National Institutes of Health (NIH Publication, revised 2011), and with the ethical standard of the 1964 Helsinki Declaration. All procedures were approved by the Laboratory Animal Ethics Committee of Jiangnan University. After 1 week of acclimatization with food and water ad libitum, the mice were randomly divided into two groups: a normal diet group and a high-fat diet group (HFD group) for 4 weeks. The high-fat-diet group was divided into groups and injected intraperitoneally with streptozotocin (STZ, 35 mg/kg in citrate buffer, pH 4.4) once three times daily to establish type 2 diabetes mellitus (T2DM). Diabetes was confirmed if fasting blood glucose was \>11.1 mmol/L for 3 consecutive days. A liver-specific AAV8 vector, coding murine SLC31A1 shRNA, under the promoter of thyroxine-binding globulin (TBG) (AAV8-TBG-shSLC31A1), was packaged in HEK293 cells. A control null particle (AAV8-TBG-shNC) packaged a non-coding plasmid. The vector was purified by cesium chloride gradient centrifugation. The information of shRNA for SLC31A1 was: 5’-CCGGCAAGTCAGCATTCGCTACAATCTCGAGATTGTAGCGAATGCTGACTTGTTTTTG-3’ (forward); 5’-AATTCAAAAACAAGTCAGCATTCGCTACAATCTCGAGATTGTAGCGAATGCTGACTTG-3’ (reverse). Seven days following STZ injection, mice were subjected to intravenous injection of AAV8-encoding shNC or shSLC31A1 (1 × 1012 infectious particles per mouse in 100 μl). These mice were fed a HFD continuously for 6 weeks. In addition, in order to further confirm the role of AMPK in the benefits of diabetes induced by the liver down-regulation of SLC31A1, we intraperitoneally injected AMPK inhibitor Dorsomorphin (30 mg kg−1 every other day) [29] for 6 weeks during hepatocyte-specific silencing of SLC31A1. After treatment, blood samples were collected by cardiac puncture. The mice were anaesthetized until reaching the endpoint criteria and euthanized. Before reaching the endpoint criteria, we ensured that the mice were fully anaesthetized by isoflurane (1·5%). Mouse euthanasia was effected by cervical dislocation. None of the mice died before reaching the indicated criteria of euthanasia. Left liver tissues were fixed in 4% formaldehyde, and the remaining liver tissues were snap-frozen in liquid nitrogen. After confirming diabetes, eligible mice were allocated to the indicated treatments at random using a computer-generated random-number sequence. Investigators who did treatment assignments were necessarily blind to the assigned interventions. Investigators assessing histology, measuring images and performing biochemical assays were blind to the group identity.

### 2.3 Intraperitoneal glucose tolerance test and insulin tolerance test (IPGTT) and insulin tolerance test (ITT)

IPGTT and ITT tests were performed to test glucose tolerance and insulin resistance, as we have previously described [30,31]. Briefly, mice were fasted for 6 h before ITT and overnight before GTT. They were given an intraperitoneal injection of insulin (0.75 U/kg) or glucose (2.0 g/kg), respectively. Blood glucose levels were measured at 0, 15, 30, 60, and 120 min after injection by a glucose monitor.

### 2.4 Detection of homeostatic model assessment-insulin resistance (HOMA-IR)

For fasting serum insulin (mU/l), ELISA kit was used. Insulin resistance was assessed using the homeostatic model assessment (HOMA-IR) which was calculated as glucose (mmol/l) × insulin (mIU/L)/22.5 as previously described [32].

### 2.5 Measurement of biochemical parameters

Whole blood was spun to give serum (3000 rpm for 10 min). Triglyceride (TG), total cholesterol (TC) and non-esterified fatty acids (NEFAs) levels were determined following test kit instructions.

### 2.6 Histological analysis

Liver samples were fixed, paraffin-embedded and sectioned (5 μm). Hematoxylin and eosin (H&E) staining was used for assessment of steatosis and Oil Red O staining was performed on frozen sections to highlight the lipid droplets. After section dehydration in absolute ethanol, sections were sealed and examined under a microscope. Sections were PAS-stained by fixing the sections with periodic acid for 20 min in the dark, then they were washed three times in deionized water. The sections were then incubated with 0.5% sodium bisulfite in 0.05 M HCl for 10 min, counterstained with hematoxylin and imaged using an Olympus BX50 light microscope. Hepatocellular vacuolation was quantified in the liver sections stained with H&E with ImageJ software. For each mouse, six randomly selected, non-overlapping areas from each of three non-adjacent liver sections were analysed by an investigator blinded to groups. Large vessels, bile ducts, tissue edges, folds, tears and non-tissue background were not included in the region of interest. The PAS positive magenta stain was extracted using color thresholding and the PAS positive area was calculated as a percentage of the parenchyma. Staining intensity was also expressed as mean optical density within the PAS positive area. Lipid accumulation was expressed as Oil Red O positive area to total liver area as a percentage. Six non-overlapping fields were analysed for each of three non-adjacent sections per mouse and the mean was used for one biological replicate.

### 2.7 Real-time quantitative reverse transcription PCR (RT-qPCR)

For each sample, total RNA from cell and liver tissues was extracted with Triquick reagent (Solarbio, Beijing, China), and reverse transcription into cDNA was performed with a Reverse transcription kit (Solarbio, Beijing, China). RT-qPCR was performed with 2× SYBR Green PCR master mix (Solarbio, Beijing, China) and the levels of relative mRNA expression determined, normalized to GAPDH. The sequence of primers was listed in Tables S1–2.

### 2.8 Immunohistochemistry and immunofluorescence staining

Freshly isolated liver tissues were fixed overnight in 4% paraformaldehyde, dehydrated and embedded in paraffin. Paraffin blocks were sectioned into 5 μm slices. Paraffin sections were subjected to antigen retrieval by pressure cooker incubation for 2 min in Tris-EDTA buffer (pH 9). Endogenous peroxidase was blocked by 3% H2O2, followed by a 30-min incubation at 37 °C with 10% goat serum to block non-specific-binding. Samples were blocked and incubated overnight at 4 °C with the primary antibody SLC31A1 (Proteintech, 27499-1-AP, 1:200). After washing, sections were incubated with a horseradish peroxidase (HRP)-conjugated secondary antibody at 37 °C for 30 min. Signal was developed using diaminobenzidine (DAB) at room temperature for 5 min. Photographs were taken by using Olympus BX50 microscopy. For immunofluorescence staining, antigen retrieval was done with citrate buffer by microwave. Sectioned tissues were blocked with 5% BSA (Beyotime, ST2254, Shanghai, China) at room temperature for 30 min before overnight blocking with primary antibodies against F4/80, Ly6G or Nrf2 at 4 °C. After washes in PBS for 3 times, sections were incubated with fluorescent secondary antibodies. Nuclei were counterstained with 4′,6-diamidino-2-phenylindole (DAPI). Samples were photographed by fluorescence microscope attached to Leica DM2500 microscope using the software LAS X (Leica). The results of immunohistochemistry staining were quantified as the percentage of DAB-positive area in the total parenchymal area. The mean optical density was calculated as the integral density of DAB signal on average area of the DAB-positive signal. The Immunofluorescence was quantified as background corrected mean fluorescence intensity in the tissue area of interest, and values from all the fields were averaged such that every mouse corresponded to one biological replicate.

### 2.9 Measurement of PEPCK and G6Pase activities

Activities of PEPCK and G6Pase were determined using commercial kits for the assay (Nanjing Jiancheng Bioengineering Institute, Nanjing, China). PEPCK activity was measured by detecting its reaction with oxaloacetate to produce phosphoenolpyruvate and carbon dioxide. This reaction was coupled to the conversion of NADH to NAD + by using pyruvate kinase and lactate dehydrogenase. The reaction was monitored by measuring the decrease of NADH absorption at 340 nm. The activity of G6Pase was determined by the production of NADPH from NADP at 340 nm. The enzyme activities were normalized to the protein content of each sample.

### 2.10 Evaluation of oxidative stress markers

Superoxide dismutase (SOD), glutathione peroxidase (GPx) activity, malondialdehyde (MDA) were detected with corresponding kits. Liver tissue and cells were homogenized by chilled PBS buffer solution and mixed with 2.3% KCl buffer solution. The suspension was centrifuged, and the liquid is collected for testing.

### 2.11 Determination of inflammatory factors

We measured the serum level of IL-1β, IL-6 and TNF-α by commercial ELISA kits according to the manufacturers’ instructions, which had been described previously. In short, Samples, diluent standard samples or blanks were added into a 96-well plate, pre-coated with antibodies. The haptens reacted with HRP-conjugated antibodies in samples, coincubated at 37 °C for 30 min. Substrates were terminated with stop solution, and absorbance values were measured with a plate reader (STNERGY/H4, BioTek, Vermont, USA).

### 2.12 Assessment of glucose and glycogen levels

Glucose and glycogen measurements were carried out using commercial assay kits as per the instructions of the kit manufacturers. For glucose measurement, cell culture medium was exchanged for DMEM with 2 mM sodium pyruvate and 20 mM sodium lactate (without phenol red). After incubation for 3 h, the glucose buffer was collected and detected by glucose oxidase–peroxidase assay kit (Jiancheng Bioengineering Institute, Nanjing, China). Glucose measurements were normalized to total protein. Glycogen levels were determined on the basis of its hydrolysis into an aldehyde derivative with concentrated sulfuric acid, which then reacted with anthrone to form a blue compound. The result was determined by a glycogen assay kit (Jiancheng Bioengineering Institute, Nanjing, China). Values were normalized to total protein.

### 2.13 TdT-mediated dUTP nick end labeling (TUNEL) staining

For the analysis of hepatic DNA fragmentation, sections were stained by TUNEL using a TUNEL staining kit as instructed by the manufacturer. Briefly, liver sections were permeabilized with 1% Triton X-100 for 20 min and then blocked with goat serum for 30 min at room temperature. Liver sections were further incubated with TUNEL reaction mixture at 37 °C for 2 h. Nuclei were counterstained with DAPI-containing antifade mounting medium and shown by confocal microscope. TUNEL-positive cells were considered to indicate DNA fragmentation, rather than being an indicator of a specific type of regulated cell death.

### 2.14 Western blotting

Liver tissue and cells were ground to liver homogenate in 500 μL prechilled PBS on ice, homogenate was collected by centrifugation at 12 000 rpm for 15 min and liver tissue homogenate was collected. The concentration of proteins was determined by BCA protein quantification kit. Equal amounts of protein were separated on a 10% SDS-PAGE and transferred to a membrane. After blocking non-specific proteins, the membrane was incubated overnight in a refrigerator (4 °C) with primary antibody (1:1000) (in blocking solution). The membrane was washed with PBS the next day, then it was incubated with the secondary antibody (1:1000) at room temperature for 2 h and protein signals were detected with enhanced chemiluminescence (ECL).

### 2.15 Determination of hepatic copper content

For measurement of hepatic copper content, we used a commercially available copper colorimetric assay kit (Cat. No. E-BC-K300-M, Elabscience, Wuhan, China) according to the manufacturer's instruction. Briefly, about 20 mg frozen liver tissues were washed with ice-cold phosphate buffered saline and homogenized into 180 μL double distilled water with ice-cold storage overnight at 4 °C. The homogenate was centrifuged for 10 min at 10 000 × g, and the supernatant was collected for measurement. Protein content was assayed by using a BCA protein assay kit. Copper standards of 0–60 μmol/L were freshly prepared. And then, liver supernatant or copper standard (15 μL) was added to 230 μL freshly prepared chromogenic reagent and mixed and placed at 37 °C for 5 min. Absorbance was read at 580 nm on a microplate reader. Fresh liver tissues were embedded in optimal cutting temperature compound, rapidly frozen and sectioned at a thickness of 8 μm by using a cryostat. Unfixed frozen sections were equilibrated in Hank's balanced salt solution for 10 min and incubated with 5 μM Coppersensor-1 (CS-1, HY-141511, MedChemExpress) in Hank's balanced salt solution at 37 °C for 20 min in the dark. After incubation, sections were washed three times with Hank's balanced salt solution to remove unbound probe. The nuclei were counterstained with DAPI. Sections were mounted with an aqueous antifade mounting medium and fluorescence images were acquired using a fluorescence or confocal microscope with excitation around 540–543 nm and emission collection around 555–600 nm.

### 2.16 Cell culture and transfection

HepG2 cells (American Type Culture Collection, Manassas, VA, USA) were cultured in DMEM supplemented with 10% FBS, 100 U/mL penicillin, and 100 μg/mL streptomycin. Cells were cultured at 37 °C in a humidified incubator with 5% CO_2_. Glucosamine (GlcN) induces glucose intolerance in muscle, liver and fat tissues by activating the hexosamine biosynthetic pathway [33]. To induce glucose metabolic stress *in vitro*, HepG2 cells were treated with 18 mM glucosamine for 18 h. To induce lipid overproduction *in vitro*, HepG2 cells were incubated with oleic acid (0.5 mM) for 24 h as previously depicted [34]. Cells were plated at 30–50% confluency and transfected with the control shRNA or SLC31A1 shRNA using Lipofectamine 2000 (Thermo Fisher Scientific, Carlsbad, CA, USA). 24 h after transfection, transfected cells were used for further experiments. After reaching 30–50% confluence, cells were transfected with either control shRNA or SLC31A1 shRNA using Lipofectamine 2000 (Thermo Fisher Scientific, Carlsbad, CA, USA). After 24 h, transfected cells were used for further experiments. Additionally, the phosphoinositide 3-kinase (PI3K) inhibitor LY294002 (10 μM), the Akt inhibitor MK2206 (1 μM) and the AMPK inhibitor Dorsomorphin (10 μM) were added 30 min before SLC31A1 shRNA transfection.

### 2.17 Construction of an shRNA-resistant SLC31A1 expression construct

To confirm the on-target specificity of SLC31A1 knockdown, we generated an shRNA-resistant expression construct of human SLC31A1. Human SLC31A1 full-length coding sequence (corresponding to RefSeq NM_001859.4) was cloned into the mammalian expression vector pcDNA3.1(+)-based expression plasmid (Invitrogen, Thermo Fisher Scientific, Waltham, MA, USA). The SLC31A1 shRNA target sequence was 5′-CCGAGAGAGCCTGCTGCGTAA-3′. We inserted 7 synonymous nucleotide substitution sites into this region by site-directed mutagenesis, generating shRNA-resistant sequence 5′-TCGCGAAAGTCTTCTTCGCAA-3′. The codon change was 5′-GCCCGAGAGAGCCTGCTGCGTAAG-3′ to 5′-GCTCGCGAAAGTCTTCTTCGCAAG-3′ and did not change the encoded amino acid.

### 2.18 Isolation of primary hepatic cell populations

Primary hepatocytes, Kupffer cells, hepatic stellate cells and liver sinusoidal endothelial cells were isolated from mice injected with AAV8-TBG-shNC or AAV8-TBG-shSLC31A1 by adapting the collagenase-perfusion and cell-fractionation protocols described previously [[1], [2], [3]].

All buffers and enzyme solutions used for perfusion of the liver were prewarmed to 37 °C, while cell-separation steps after perfusion were performed at 4 °C unless noted otherwise. Mice were deeply anaesthetized, and the abdominal cavity was opened under sterile conditions. The portal vein was cannulated and the inferior vena cava was cut to promote outflow. The liver was first perfused with 20 mL calcium and magnesium-free Hank's balanced salt solution supplemented with 0.5 mM EGTA and 10 mM HEPES at a flow rate of around 3–4 mL min−1. The liver was then perfused with 20–25 mL digestion buffer containing 0.5 mg/mL collagenase type IV, 5 mM CaCl_2_, 10 mM HEPES, and 0.05 mg/mL DNase I.

Once digestion was sufficient (softening and swelling of the liver), liver was excised and placed in a sterile dish filled with cold isolation medium. The liver capsule was disrupted gently and released cells were passed through a 70-μm cell strainer. The filtered cells suspension was centrifuged sequentially for differential settling of the separated fractions of hepatocyte-enriched and non-parenchymal cells fractions. Filtered cell suspension was centrifuged at 50 ×g for 3 min at 4 °C. The pellet of hepatocyte-enriched cells was collected and resuspended gently with cold isolation medium. Washing of cells was repeated thrice at 50 ×g for 3 min to remove residual non-parenchymal cells from filtered cells. Viability of cells was confirmed using trypan-blue exclusion. The isolated hepatocytes were processed immediately for RNA extraction. Kupffer cells were purified from the non-parenchymal cell fraction of liver by anti-F4/80 magnetic microbeads as per the manufacturer's instructions. Briefly, cells were resuspended in magnetic-activated cell-sorting buffer and incubated with anti-F4880 microbeads for 15 min at 4 °C. The incubation solution was collected after washing cells and eluted using an LS separation column loaded on a magnetic separator. The LS column was washed three times, removed from the magnetic separator and the retained F4/80-positive cells collected are eluted. The collected cells were directly used for RNA extraction. Liver sinusoidal endothelial cells were enriched from the non-parenchymal cell fraction of the liver with anti-CD146 magnetic microbeads. Cells were incubated with anti-CD146 microbeads for 15 min at 4 °C, washed and loaded on an LS column placed in a magnetic separator. The LS column was washed three times and removed from the magnetic field, with the retained CD146-positive cells collected then eluted. Hepatic stellate cells were isolated by a modified method for pronase–collagenase perfusion and density-gradient procedure. Briefly, the liver was perfused with an EGTA-containing buffer, pronase solution and collagenase solution. After excision of the liver, the liver was minced and then resuspended in another round of digestion in a mixture containing pronase, collagenase and DNase I, which was incubated at 37 °C for 30 min. Digested cells suspension was filtered through a 70-μm cell strainer and washed by centrifugation. The cell pellet was resuspended in Gey's balanced salt solution and mixed with Nycodenz solution. After density-gradient centrifugation, the low-density cell layer at the interface of the density gradient was collected, washed and used immediately for RNA extraction.

### 2.19 Measurement of AMP/ATP ratio using commercial kits

Cell level of AMP and ATP was determined with commercially available assay kits (MAK473 for ATP, Sigma-Aldrich; ABIN512776 for AMP, ANTIBODIES ONLINE) according to manufacturer's instructions [35]. Briefly, HepG2 cells were plated into 96-well white opaque plates and treated as mentioned above. After treatment, cells were lysed with provided lysis buffer, the level of AMP and ATP was determined using bioluminescence-based assay. Luminescent signals were recorded in a microplate reader (SpectraMax i3x, Molecular Devices). For each well, the ratio of AMP to ATP was obtained by dividing the signals of AMP by those of ATP.

### 2.20 Statistical analysis

Results are given as mean ± standard deviation (SD). All statistical analyses were done using the software package GraphPad Prism 8.0. Normality was tested by using the Shapiro–Wilk test and homogeneity of variance by the Brown–Forsythe test. For comparisons between two groups, an unpaired two-tailed Student's *t*-test was used if the data followed normal distribution and were homoscedastic, and a Welch *t*-test was used if homoscedasticity was not present; if the data were not normally distributed, the Mann–Whitney *U* test was used. For comparisons between multiple groups, a one-way analysis of variance (ANOVA) followed by Bonferroni-adjusted post-hoc comparisons or two-way ANOVA followed by Tukey multiple comparison test (HSD) post-hoc comparison (which were all parametric tests) was used if the parametric assumptions were satisfied. If homoscedasticity was not satisfied, use of Welch ANOVA with an appropriate multiple-comparison procedure was appropriate and the Kruskal–Wallis test with Dunn multiple-comparison test post hoc was used for non-normally distributed data. IPGTT and ITT time-course data were analysed by linear mixed-effects model analysis, with treatment group, time and their interaction included as fixed effects, and mouse identity as random intercept. When a significant group-by-time interaction effect was observed, multiplicity corrected comparisons were done to compare groups at prespecified time points. AUCs were calculated for each mouse and were analysed separately by two-way ANOVA. A two-sided *P*-value of < 0.05 was considered significant.

## 3 Results

### 3.1 SLC31A1 expression and hepatic copper accumulation are increased in T2D mice

**Fig. 1:**
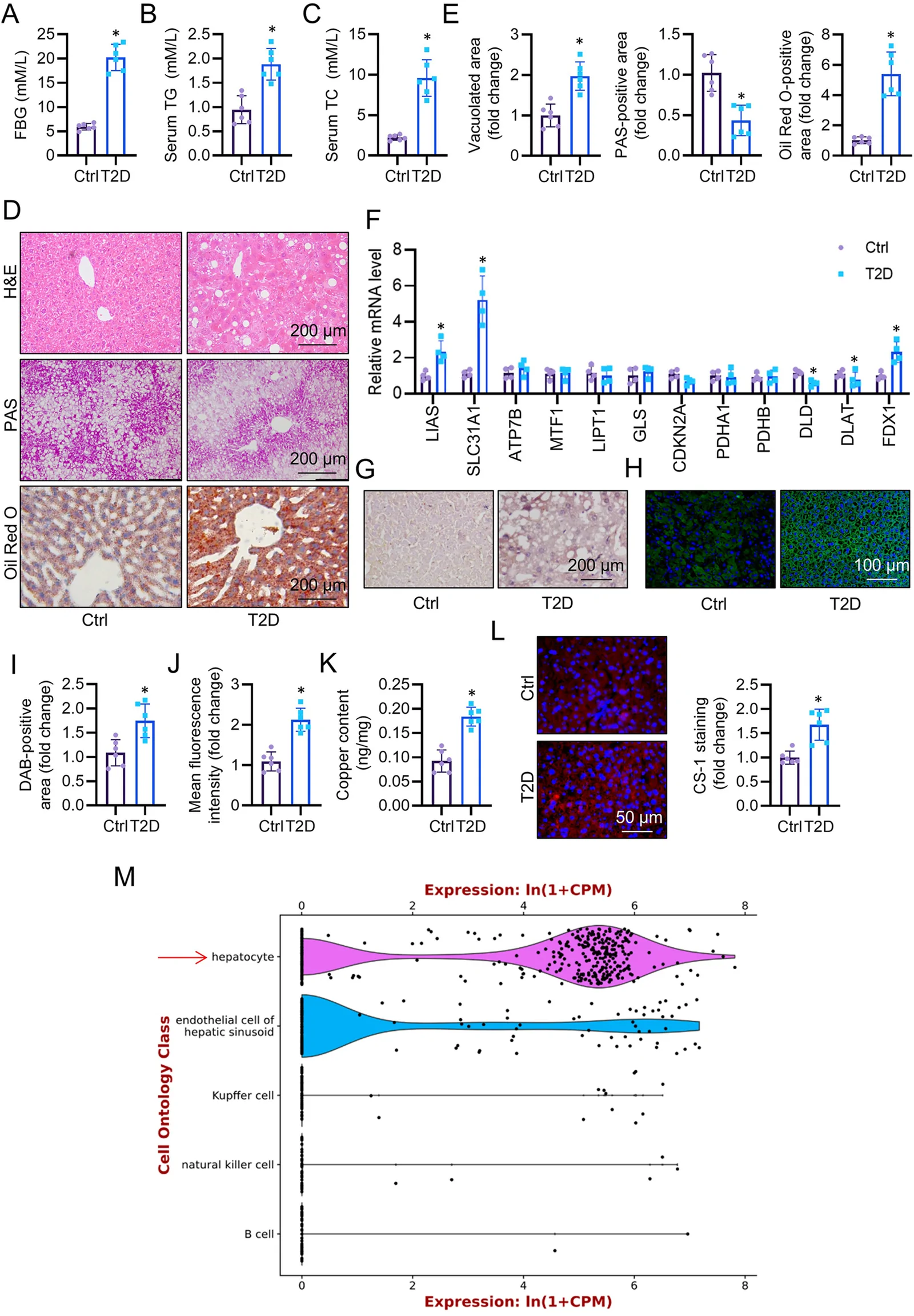
**Hepatic SLC31A1 expression and copper accumulation are increased in T2D mice.** (**A**) Fasting blood glucose (FBG) level. (**B**) Serum TG level. (**C**) Serum TC level. (**D**) H&E staining, PAS staining, and Oil Red O staining of liver sections. (**E**) Quantitation of the tissue histological steatosis, PAS-positive area, and Oil Red O-positive area of the liver sections. (**F**) Relative mRNA level of LIAS, SLC31A1, ATP7B, MTF1, LIPT1, GLS, CDKN2A, PDHA1, PDHB, DLD, DLAT, and FDX1. (**G**) Immunohistochemistry for SLC31A1. (**H**) Immunofluorescence staining for SLC31A1. (**I**) Quantification of hepatic SLC31A1 expression. SLC31A1 immunoreactivity was expressed as DAB-positive area. (**J**) Quantification of hepatic SLC31A1 expression. Fluorescence is expressed as as background-corrected mean fluorescence intensity. (**K**) Copper content in the livers. (**L**) CS-1 staining of the liver sections. Quantified CS-1 fluorescent intensity was increased significantly in the T2D group. (**M**) The distribution of SLC31A1 in live mouse samples from the Tabula Muris database. n = 4-6 for per group. For comparisons between two groups, an unpaired two-tailed Student's *t*-test was performed. *P < 0.05 *vs*. Control (Ctrl).

Previous studies have implicated the dysregulation of copper metabolism and cuproptosis-associated pathways in diabetes [[36], [37], [38]]. First, we analysed the mRNA expression of genes for copper homeostasis and cuproptosis, including LIAS, SLC31A1, ATP7B, MTF1, LIPT1, GLS, CDKN2A, PDHA1, PDHB, DLD, DLAT, and FDX1 in the livers of control and T2D mice. Compared with control mice, T2D mice developed hyperglycaemia and high serum total cholesterol (TC) and triglyceride (TG) levels were detected (Fig. 1A–C). Hematoxylin and eosin (H&E) staining showed that the percentage of vacuolated liver area in T2D mice was higher than that of control mice (Fig. 1D–E). Periodic-Acid-Schiff (PAS) staining demonstrated heavily magenta-labelled glycogen deposits in liver from control mice but PAS staining was observed at a lower extent in the livers from T2D mice (Fig. 1D–E). Percentages of Oil Red O-positive area in T2D mice were much higher, showing increased accumulation of hepatic neutral-lipid in T2D mice (Fig. 1D–E). Real-time quantitative PCR showed expression profiles of five different genes in the livers of T2D mice (Fig. 1F). Specifically, the mRNA expression of LIAS, SLC31A1 and FDX1 increased in the livers of T2D mice, and the mRNA levels of the DLAT and DLD genes were severely suppressed (Fig. 1F). Particularly, SLC31A1 caught our attention with a nearly five times increased mRNA expression level (Fig. 1F). Immunohistochemistry and immunofluorescence showed an obvious up-regulation of SLC31A1 protein expression in livers of T2D mice in comparison with normal mice (Fig. 1G–J). Consistently, the contents of copper in the livers of T2D mice tended to be higher (Fig. 1K). CopperSensor-1 (CS-1) is a specific fluorescent probe of intracellular copper, and it can be combined with metal copper content to detect metal.

Support, the fluorescence staining showed enhanced Cu in the livers of T2D mice (Fig. 1L). To ascertain which hepatic cell type gave rise to the upregulation of SLC31A1 in mice livers, we first checked the expression of SLC31A1 in mouse livers using the Tabula Muris database. Single cell sequencing of mouse liver tissues showed that SLC31A1 was mainly expressed in hepatocytes (Fig. 1M). This was consistent with our observation that the HUMAN PROTEIN ATLAS (https://www.proteinatlas.org/), an online database that maps the expression of proteins in human cells, tissues and organs, again indicated predominant expression of SLC31A1 in hepatocytes (Fig. S1A–B). Therefore, the human hepatocellular carcinoma cells (HepG2) cells treated with GlcN or OA were used to simulate disordered glucose and lipid metabolism in diabetes *in vitro*.

### 3.2 Hepatocyte-specific SLC31A1 knockdown attenuates hyperglycemia and hepatic insulin resistance in T2D mice

**Fig. 2:**
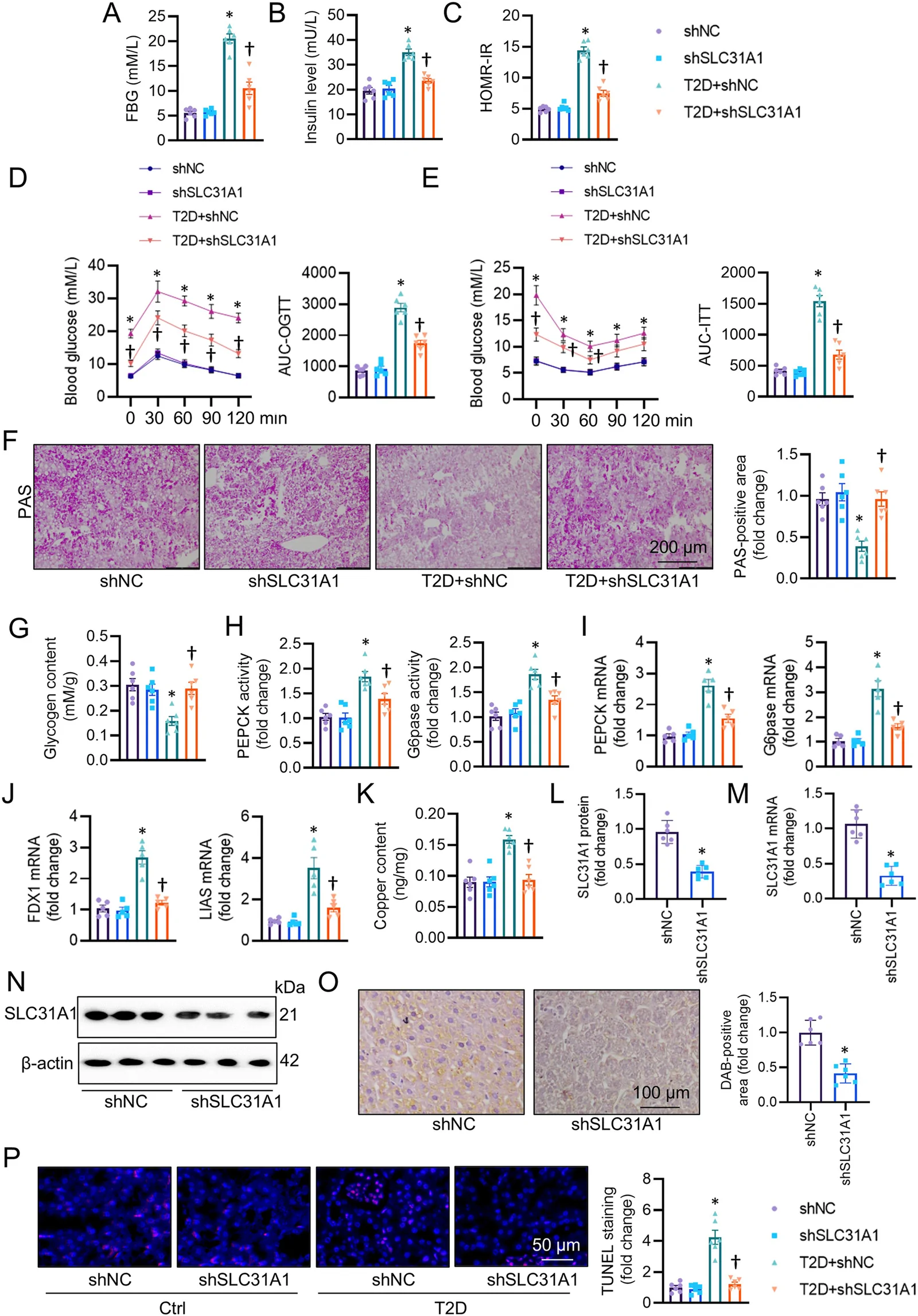
**Hepatocyte-specific SLC31A1 knockdown alleviates hyperglycemia and hepatic insulin resistance in T2D mice.** (**A**) FBG levels. (**B**) Serum insulin levels. (**C**) HOMR-IR. (**D**) IPGTT and area under curve (AUC) of IPGTT. (**E**) ITT and area under curve (AUC) of ITT. (**F**) PAS staining of sections of the liver and quantitative analyses of PAS-positive area. (**G**) Glycogen contents. (**H**) PEPCK activity and G6pase activity. (**I**) Relative mRNA levels of PEPCK and G6pase. (**J**) Relative mRNA levels of FDX1 and LIAS. (**K**) Copper contents in the liver. (**L,N**) Representative blot and relative quantitative analysis of SLC31A1. (**M**) Relative mRNA level of SLC31A1. (**O**) Immunohistochemistry for SLC31A1 and quantitative analyses. (**P**) Representative TUNEL staining and quantification results of TUNEL-positive cells in liver sections. n = 5 to 6 for each group. For comparisons among multiple groups, two-way ANOVA followed by Tukey's HSD post-hoc comparisons was used (A-K). For comparisons between two groups, an unpaired two-tailed Student's *t*-test was used (L-P). *P < 0.05 *vs*. shNC, †*P* < 0.05 *vs*. T2D + shNC.

Upon detailed characterization of the possible role of SLC31A1 in diabetes-related function deterioration in the liver, we specifically knocked down SLC31A1 under the trigger of TBG promoter (AAV8-TBG-SLC31A1 shRNA) in T2D mice. We examined the metabolic parameters in a T2D model of HFD/STZ-induced mice. Fasting blood glucose (FBG) levels were higher in untreated diabetic mice, whereas hepatocyte-specific SLC31A1 knockdown increased hepatic glucose metabolism in the livers (Fig. 2A). The state of hyperinsulinemia was markedly alleviated by SLC31A1 downregulation in the livers (Fig. 2B). Hepatocyte-specific SLC31A1 knockdown decreased HOMA-IR to near normal levels (Fig. 2C). IPGTT showed improvement in glucose disposal capacity in SLC31A1 downregulation-treated groups than untreated diabetic control groups (Fig. 2D), with parallel improvement in insulin sensitivity observed by insulin tolerance test (Fig. 2E). By PAS staining, the hepatic glycogen storage was reduced in T2D mice, and this reduction was alleviated by AAV8-TBG-induced SLC31A1 gene knockdown (Fig. 2F). Consistent with this, our biochemistry data showed that SLC31A1 gene knockdown rescued the reduced glycogen in the liver of T2D mice (Fig. 2G).

As dysregulated hepatic gluconeogenesis is a pathophysiologic hallmark of insulin resistance and glucose metabolic disorders [33], we next explored whether SLC31A1 contributes to diabetes through regulation of hepatic gluconeogenesis. The expression and activity of phosphoenolpyruvate carboxykinase (PEPCK) and glucose-6-phosphatase (G6Pase), the two enzymes principally involved in gluconeogenesis, were therefore determined. As reported before [33,39], we found a higher activity and mRNA levels of PEPCK and G6Pase in T2D mice and such activities were largely abrogated by SLC31A1 downregulation (Fig. 2H–I). Strikingly, hepatic SLC31A1 knockdown re-established the gene expression of cuproptosis markers FDX1 and LIAS and decreased hepatic Cu concentration in T2D mice (Fig. 2J–K). AAV8-induced downregulation of SLC31A1 in livers was verified by Western blotting, RT-PCR, and immunohistochemistry (Fig. 2L–O). We also determined the distribution of AAV8-TBG-induced SLC31A1 knockdown in other tissues. SLC31A1 expression was measured in liver and in the main extrahepatic tissues including heart, kidney, lung, spleen, skeletal muscle, adipose tissue. A reduction in SLC31A1 expression was observed in liver, but no significant reduction was observed in the extrahepatic tissues analysed (Fig. S2A). To evaluate the intrahepatic cell-type specificity of knockdown of SLC31A1 by AAV8-TBG directly, we isolated the four major cell populations in the liver (hepatocyte, Kupffer cell, hepatic stellate cell and liver sinusoidal endothelial cell), from mice treated with AAV8-TBG-shNC or AAV8-TBG-shSLC31A1. AAV8-TBG-shSLC31A1 greatly reduced SLC31A1 expression in primary hepatocytes, but not in Kupffer cells, hepatic stellate cells and liver sinusoidal endothelial cells, from treated mice (Fig. S2B). These observations provided direct evidence that the knockdown induced by AAV8-TBG vector was highly enriched in hepatocytes. TUNEL staining demonstrated further that hepatic SLC31A1 knockdown in hepatocytes attenuates hepatic DNA fragmentation in T2D mice, thereby reducing the level of hepatocellular injury (Fig. 2P).

### 3.3 Hepatocyte-specific SLC31A1 knockdown attenuates hepaosteatosis in T2D mice

**Fig. 3:**
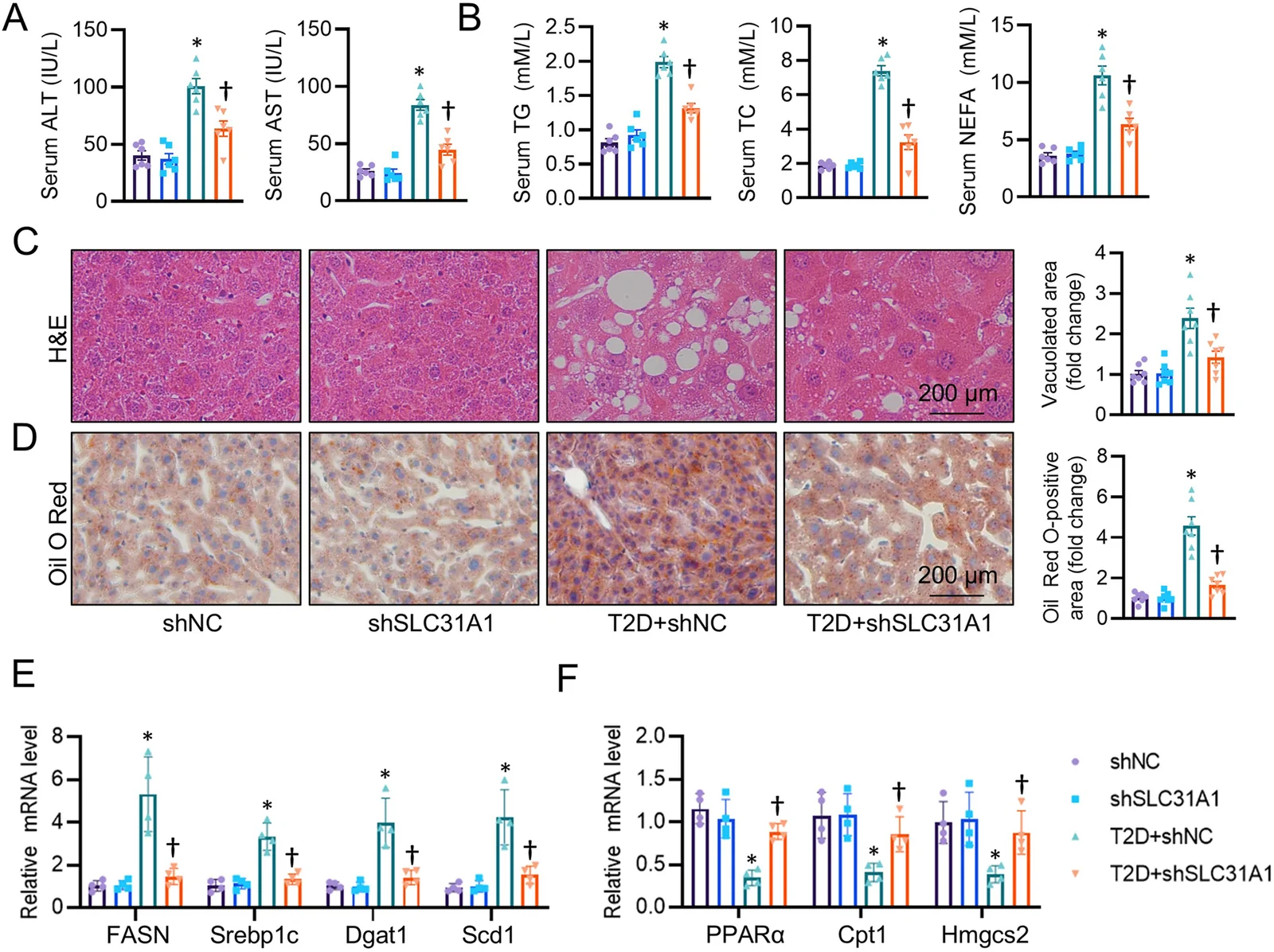
**Hepatocyte-specific SLC31A1 knockdown attenuates hepaosteatosis in T2D mice.** (**A**) Serum ALT and AST. (**B**) Serum TG, TC, and NEFA levels. (**C**) H&E staining of liver sections and quantitative analyses of the vacuolated area of liver sections. (**D**) Oil Red O staining of liver sections and quantitative analyses of Oil Red O-positive area of liver sections. (**E**) Relative mRNA levels of *FASN*, *Srebp1c*, *Dgat1*, and *Scd1*. (**F**) Relative mRNA levels of *PPARα*, *Cpt1*, and *Hmgcs2*. n = 4-6 for each group. For comparisons among multiple groups, two-way ANOVA followed by Tukey's HSD post-hoc comparisons was used. *P < 0.05 *vs*. shNC, †*P* < 0.05 *vs*. T2D + shNC.

Next, we examined the effects of SLC31A1 on the liver function and blood lipid levels of T2DM mice. We detected serum ALT, AST, TG, TC and NEFA levels in mice. Compared with normal mice, the serum levels of ALT, AST, TG, TC and NEFA in T2DM group mice were dramatically increased. After 6 weeks of SLC31A1 downregulation, the level of serum ALT, AST, TG, TC and NEFA remarkably decreased (Fig. 3A–B). There were disordered liver cells and obvious vacuolar distending in T2DM mouse liver tissues (H&E staining). However, the liver cell-specific knockdown of SLC31A1 decreased the vacuole of adipose tissue, SLC31A1 deficiency could obviously alleviate liver damage of T2D mice (Fig. 3C). Consistent with this, knockdown of hepatocyte-specific SLC31A1 decreased lipid deposition in T2D mice (Fig. 3D). As displayed in Fig. 3E–F, T2D mice had higher lipogenic genes (*FASN*, *Srebp1c*, *Dgat1*, and *Scd1*) and lower fatty acid oxidation (FAO) genes (*PPARα*, *Cpt1*, and *Hmgcs2*) in the livers. In contrast, hepatocyte-specific SLC31A1 knockdown reversed such effects in T2D mice (Fig. 3E–F).

### 3.4 Effects of SLC31A1 downregulation on glycogen content and lipid accumulation in cultured hepatocytes

**Fig. 4:**
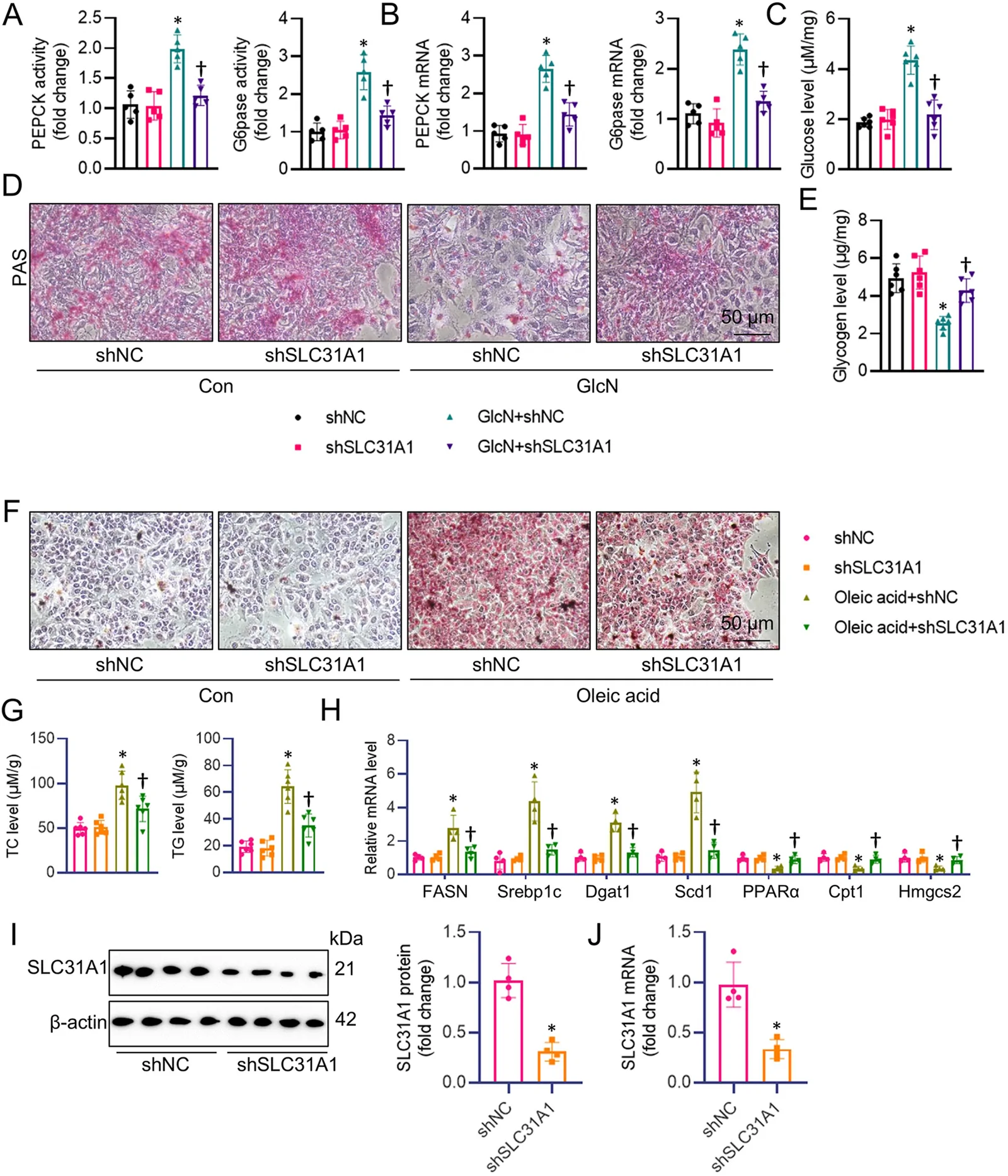
**Effects of SLC31A1 downregulation on glycogen content and lipid accumulation in hepatocytes.** (**A**) PEPCK activity and G6pase activity. (**B**) Relative mRNA levels of PEPCK and G6pase. (**C**) Glucose level. (**D**) PAS staining of hepatocytes. (**E**) Glycogen content. (**F**) Oil O Red staining of hepatocytes. (**G**) TC and TG contents. (**H**) Relative mRNA levels of *FASN*, *Srebp1c*, *Dgat1*, *Scd1*, *PPARα*, *Cpt1*, and *Hmgcs2*. (**I**) Representative blots and quantitative analysis of SLC31A1. (**J**) Relative mRNA level of SLC31A1. n = 4-6 for each group. For comparisons among multiple groups, two-way ANOVA followed by Tukey's HSD post-hoc comparisons was used (A-H). For comparisons between two groups, an unpaired two-tailed Student's *t*-test was used (I-J). *P < 0.05 *vs*. shNC, †*P* < 0.05 *vs*. GlcN + shNC or Oleic acid + shNC.

Then, we studied whether SLC31A1 knockdown reduced GlcN-induced changes in glucose production and glycogen metabolism in cultured hepatocytes, HepG2 cells. We found that SLC31A1 knockdown obviously blocked the effects of GlcN on the activities and mRNA levels of PEPCK and G6pase and the glucose formation in cultured hepatocytes (Fig. 4A–C). PAS staining showed that GlcN treatment reduced glycogen content in HepG2 cells, and knockdown of SLC31A1 attenuated the reduction (Fig. 4D). Consistently, biochemical assay showed that knockdown of SLC31A1 restored glycogen content in HepG2 cells cultured with GlcN (Fig. 4E).

Furthermore, we tested the role of deficiency of SLC31A1 on lipid accumulation in hepatocytes challenge by oleic acid. Oil Red O staining showed that oleic acid challenge increased lipid accumulation in hepatocytes (Fig. 4F). This finding was further confirmed by the measurement of TG and TC levels (Fig. 4G). By contrast, knockdown of SLC31A1 abolished these effects (Fig. 4F–G). Most interestingly, transcriptional levels of the lipogenic genes (FASN, Srebp1c, Dgat1 and Scd1) were upregulated, but those of FAO-related genes (PPARα, Cpt1 and Hmgcs2) were downregulated in hepatocytes after oleic acid treatment (Fig. 4H). Such effects were dramatically alleviated by downregulation of SLC31A1 (Fig. 4H). SLC31A1 shRNA caused obvious decreases of protein and mRNA levels of SLC31A1 in hepatocytes (Fig. 4I–J).

We also performed an on-target rescue experiment by using an shRNA-resistant SLC31A1 construct with silent nucleotide substitutions for the shRNA-recognition sequence. Re-expression of the shRNA-resistant SLC31A1 in SLC31A1-knockdown hepatocytes restored SLC31A1 protein expression and mRNA levels in GlcN-treated cells (Fig. S3A–B). Importantly, SLC31A1 re-expression largely abolished the beneficial effects of SLC31A1 knockdown on glucose production and glycogen content (Fig. S3C). Consistently, SLC31A1 re-expression (Fig. S3D–E) prevented the effects of SLC31A1 knockdown on lipid accumulation (Fig. S3F) in oleic acid-stimulated cells. These data strengthed the on-target specificity of the SLC31A1-knockdown phenotype. We next assessed whether the effects we observed were SLC31A1-dependent with regard to cellular copper availability. Treatment with a copper chelator Tetrathiomolybdate [40] (TTM, 10 μM) reduced the levels of intracellular copper (Fig. S4A–B) and phenocopied the effects of SLC31A1 knockdown on the level of glucose and TG (Fig. S4C–D). By contrast, treatment with CuCl_2_ partially reversed the effects of SLC31A1 knockdown on the levels of glucose and TG (Fig. S4E–F). These findings support a role of changed copper availability in the effect of SLC31A1.

### 3.5 Hepatocyte-specific SLC31A1 knockdown inhibits inflammation and oxidative stress in T2D mice

**Fig. 5:**
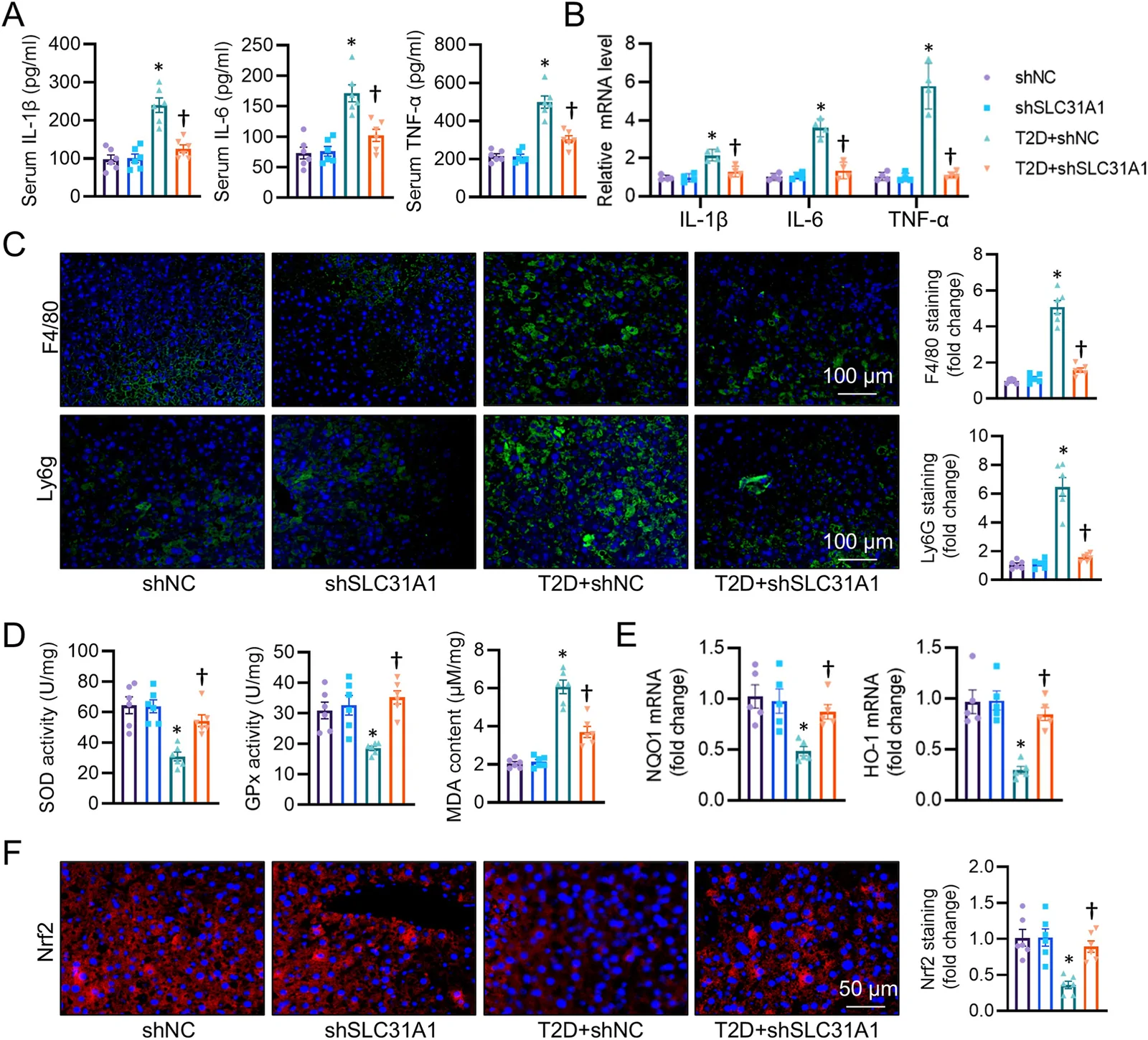
**Hepatocyte-specific SLC31A1 knockdown inhibits inflammation and oxidative stress in T2D mice.** (**A**) Serum levels of IL-1β, IL-6 and TNF-α. (**B**) Relative mRNA levels of IL-1β, IL-6 and TNF-α. (**C**) F4/80 and Ly6g staining of liver section and quantitative analyses. (**D**) SOD activity, glutathione peroxidase (GPx) activity and MDA content. (**E**) Relative mRNA levels of NQO1 and HO-1. (**F**) Nrf2 staining of liver section and quantitative analyses. n = 4-6 for each group. For comparisons among multiple groups, two-way ANOVA followed by Tukey's HSD post-hoc comparisons was used. *P < 0.05 *vs*. shNC, †*P* < 0.05 *vs*. T2D + shNC.

Reactive oxygen species (ROS) generation and inflammation caused by longstanding hyperglycemia are well-established effects of T2D and are key features of its pathogenesis [[41], [42], [43]]. Results showed that serum IL-1β, IL-6 and TNF-α levels were obviously elevated in T2D mice, but were prevented by hepatocyte-specific SLC31A1 knockdown (Fig. 5A). Together, hepatocyte-specific SLC31A1 knockdown inhibited the mRNA level of IL-1β, IL-6 and TNF-α in the livers of T2D mice (Fig. 5B). Consistently, accumulation of macrophage (f4/80 staining) and neutrophils (Ly6g staining) was observed in the livers of T2D mice, and was reversed by hepatocyte-specific SLC31A1 knockdown (Fig. 5C). In T2D mice, the levels of antioxidant enzyme activities (SOD and GPx) were remarkably downregulated, and MDA contents were remarkably elevated (Fig. 5D). Also, hepatocyte-specific knockdown of SLC31A1 restored the activity of SOD and GPx, and reduced the MDA contents of the livers of T2D mice (Fig. 5D).

Nrf2 is a critical regulator of antioxidant genes expression and NQO1, HO-1 are downstream proteins of Nrf2 that are regulated by Nrf2 [44,45]. We next sought to explore the mechanism of the reduction in oxidative stress by hepatocyte-specific knockdown of SLC31A1 in the livers. Over-sugar downregulated the mRNA abundance of NQO1 and HO-1 in the livers, whereas this was restored by hepatocyte-specific knockdown of SLC31A1 (Fig. 5E). In addition, knockdown of SLC31A1 increased the abundance of Nrf2 in the livers of T2D mice (Fig. 5F).

### 3.6 SLC31A1 contributes hepatic glucose and lipid metabolic disturbance by inactivating the AMPKα1 pathway

**Fig. 6:**
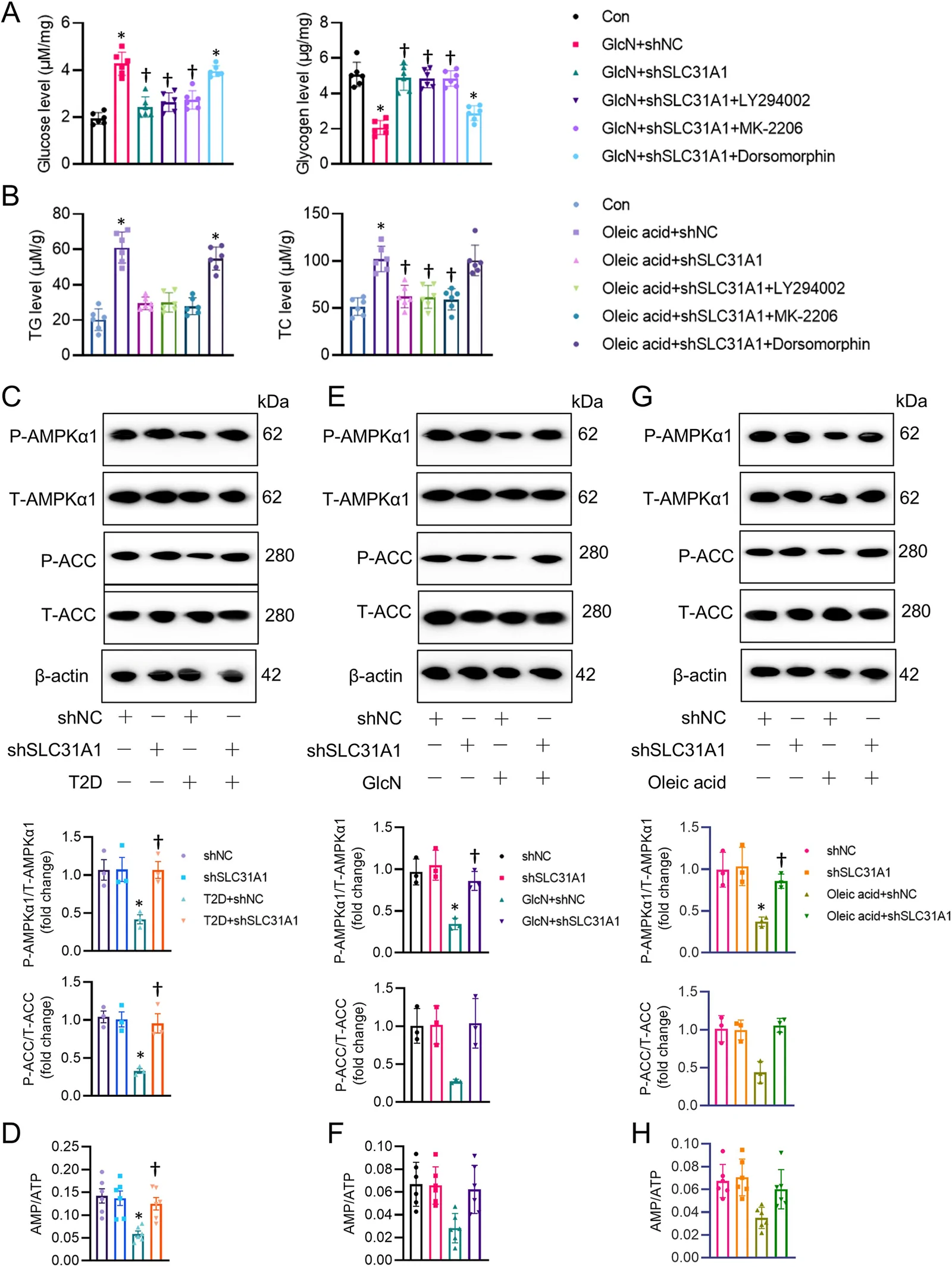
**SLC31A1 contributes hepatic glucose and lipid metabolic disturbance by inactivating the AMPKα1 pathway.** (**A**) Glucose and glycogen levels in hepatocytes. (**B**) TG and TC contents in hepatocytes. (**C**) Representative blots and quantitative analysis of P-AMPKα1 and P-ACC in the liver of T2D mice. (**D**) AMP/ATP ratio in liver of T2D mice. (**E**) Representative blots and quantitative analysis of P-AMPKα1 and P-ACC in GlcN-treated hepatocytes. (**F**) AMP/ATP ratio in GlcN-treated hepatocytes. (**G**) Representative blots and quantitative analysis of P-AMPKα1 and P-ACC in oleic acid-treated hepatocytes. (**H**) AMP/ATP ratio in oleic acid-treated hepatocytes. n = 3-6 for each group. For comparisons among multiple groups, one-way ANOVA followed by Bonferroni-adjusted post-hoc comparisons was used (A-B). For comparisons among multiple groups, two-way ANOVA followed by Tukey's HSD post-hoc comparisons was used (C-H). *P < 0.05 *vs*. Con or shNC, †*P* < 0.05 *vs*. T2D + shNC, GlcN + shNC or Oleic acid + shNC.

Diabetes-induced liver dysfunction is a result of chronic metabolic stress, oxidative damage and altered signaling pathways. Among them, PI3K/Akt and AMPK pathways are important factors in maintaining liver homeostasis and metabolic equilibrium [46,47]. Therefore, we next tested whether SLC31A1 participated in diabetes-induced liver dysfunction through PI3K/Akt and AMPK pathways. The influence of downregulation of SLC31A1 on glucose production and glycogen synthesis was reversed by AMPK inhibitor Dorsomorphin, but not PI3K inhibitor LY294002 or Akt inhibitor MK-2206 (Fig. 6A). Similarly, AMPK inhibitor Dorsomorphin reversed the effects of downregulation of SLC31A1 on TG and TC production in hepatocytes after the addition of oleic acid (Fig. 6B). Western blotting indicated that knockdown of SLC31A1 increased phosphorylation of canonical AMPK downstream substrate ACC and recovered AMP/ATP ratio in the livers of T2D mice (Fig. 6C–D). Similar results were observed in GlcN- and oleic acid-treated cells (Fig. 6E–H).

As AMPK has two catalytic α-subunits that may have distinct metabolic activities, we next studied AMPKα1 and AMPKα2 separately [48,49]. GlcN and oleic acid stimulation also reduced phosphorylation of AMPKα2 in hepatocytes (Fig. S5A–B). Interestingly, AMPKα2 phosphorylation was not affected by SLC31A1 knockdown (Fig. S5A–B). To evaluate the role of each AMPK catalytic subunit genetically, AMPKα1 or AMPKα2 was individually depleted by specific siRNAs (Fig. S5C). Depletion of AMPKα1 abolished the inhibitory action of SLC31A1 knockdown on glucose production and intra-cellular TG accumulation (Fig. S5D–E). By contrast, AMPKα2 knockdown did not have a significant effect on those metabolic changes (Fig. S5D–E).

**Fig. 7:**
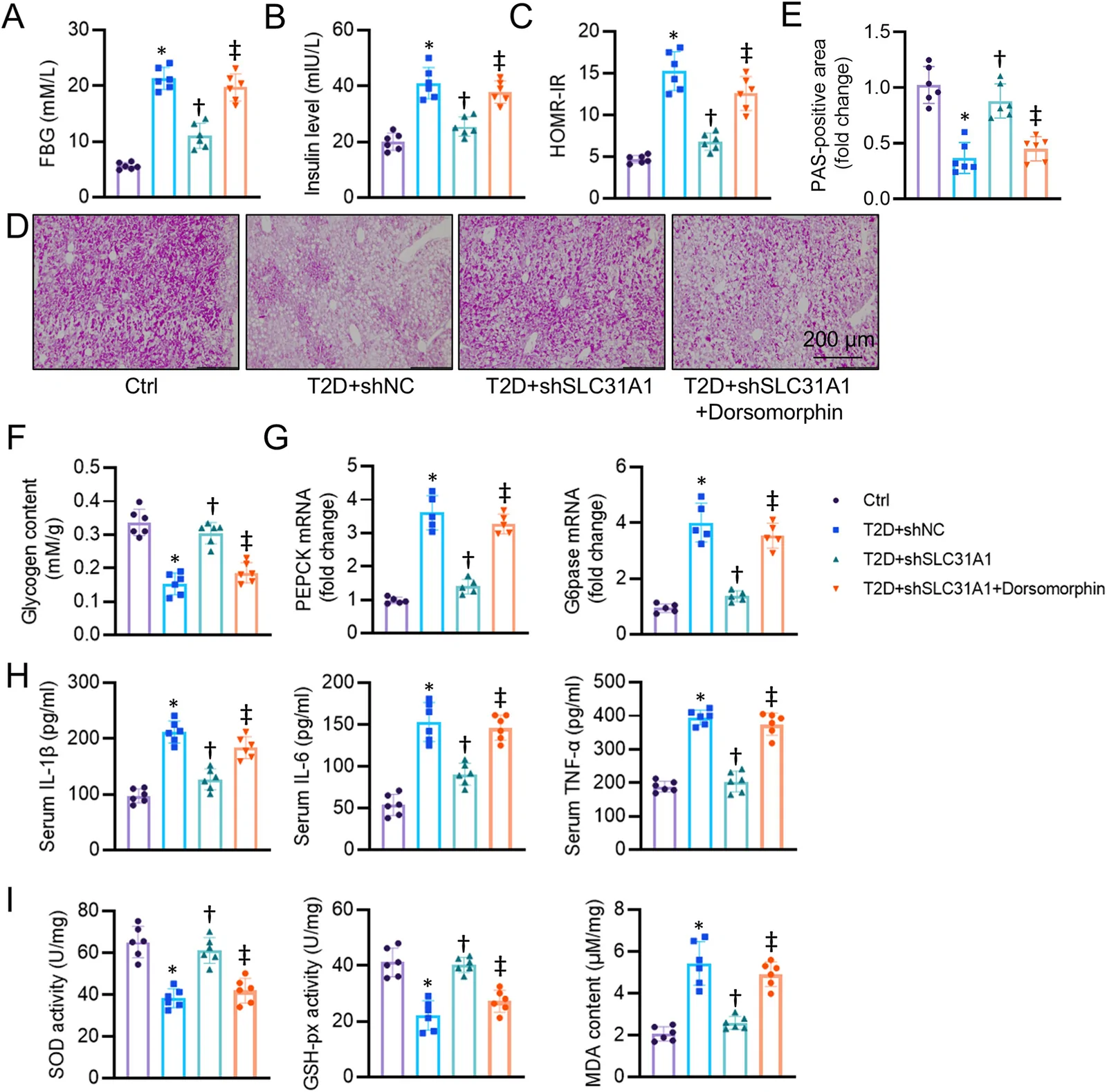
**AMPK inhibition prevents the effects of SLC31A1 downregulation on hepatic insulin resistance, oxidative stress, and inflammation in T2D mice.** (**A**) FBG level. (**B**) Serum insulin level. (**C**) HOMR-IR. (**D**) PAS staining of liver sections. (**E**) Quantitative analyses of PAS staining. (**F**) Contents of glycogen. (**G**) Relative mRNA levels of PEPCK and G6pase. (**H**) Contents of serum IL-1β, IL-6 and TNF-α. (**I**) SOD activity, glutathione peroxidase (GPx) activity and MDA contents. n = 5-6 for each group. For comparisons among multiple groups, one-way ANOVA followed by Bonferroni-adjusted post-hoc comparisons was used. *P < 0.05 *vs*. Ctrl, †*P* < 0.05 *vs*. T2D + shNC, ‡*P* < 0.05 *vs*. T2D + shSLC31A1.

**Fig. 8:**
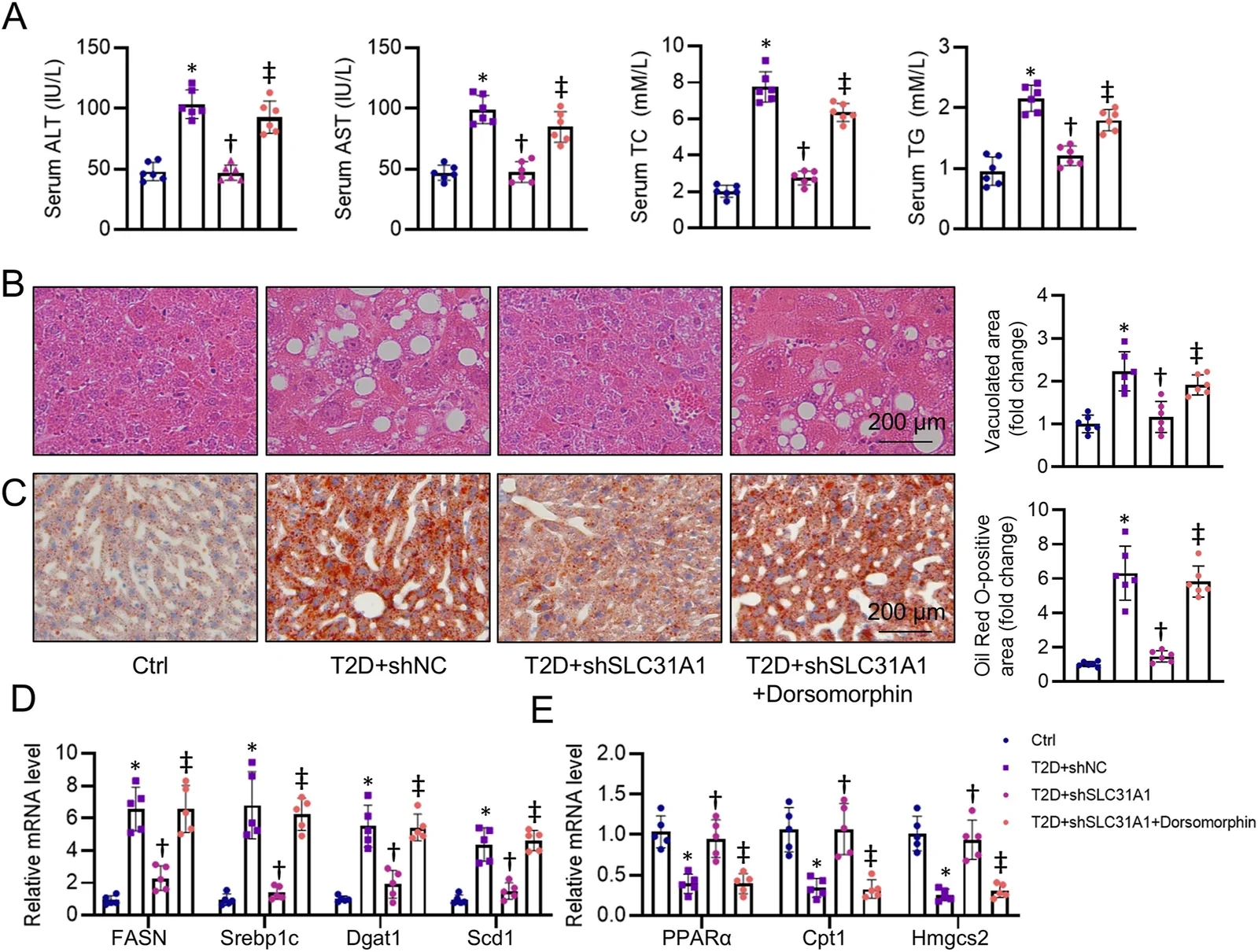
**AMPK inhibition prevents the effects of SLC31A1 downregulation on hepatic lipid accumulation in T2D mice.** (**A**) Serum ALT, AST, TC, and TG levels. (**B**) H&E staining and assessment of liver sections and quantitative analyses. (**C**) Oil Red O staining and assessment of liver sections and quantitative analyses. (**D**) Relative mRNA levels of *FASN*, *Srebp1c*, *Dgat1*, and *Scd1*. (**E**) Relative mRNA levels of *PPARα*, *Cpt1*, and *Hmgcs2*. n = 5-6 in each group. For comparisons among multiple groups, one-way ANOVA followed by Bonferroni-adjusted post-hoc comparisons was used. *P < 0.05 *vs*. Ctrl, †*P* < 0.05 *vs*. T2D + shNC, ‡*P* < 0.05 *vs*. T2D + shSLC31A1.

To further confirm SLC31A1 dependence of AMPK inactivation, we used Dorsomorphin (AMPK inhibitor) to treat SLC31A1-deficient T2D mice. As mentioned above, we observed that downregulation of hepatocyte SLC31A1 had enhanced effects on fasting blood glucose and insulin resistance, HOMA-IR in T2D mice, which were substantially blocked by Dorsomorphin (Fig. 7A–C). Inactivation of AMPK reversed the effects of downregulation of SLC31A1 on glycogen contents of livers in T2D mice (Fig. 7D–F). The suppression effects of downregulation of SLC31A1 on activities of PEPCK and G6pase in livers were reversed by Dorsomorphin (Fig. 7G). Also, the anti-inflammatory and anti-oxidation effects of downregulation of SLC31A1 on T2D mice were reversed by Dorsomorphin (Fig. 7H–I). Improvement of hepatic lipid metabolism by hepatocyte-specific SLC31A1 knockdown was reverted by Dorsomorphin (measurement of serum ALT, AST, TG and TC, H&E staining and Oil O red staining, Fig. 8A–C) and Dorsomorphin reversed the regulation of SLC31A1 deficiency on lipogenic genes and FAO-related genes tremendously (Fig. 8D–E).

## 4 Discussion

For the present study, we observed an increase of SLC31A1 expression and copper content in the liver of T2D mice. Hepatocyte-specific SLC31A1 knockdown ameliorated hyperglycemia and insulin resistance, hepatic glucose metabolism, lipid accumulation, oxidative stress, inflammation and liver injury. SLC31A1 knockdown ameliorated AMPK phosphorylation, and pharmacological inhibition of AMPK partially reversed the beneficial effect on AMPK phosphorylation. Altogether, these data identified the hepatocyte SLC31A1 as a contributor to hepatic Cu dyshomeostasis and metabolic dysfunction in T2D mice and revealed that this effect may be mediated, at least in part, via inhibition of AMPK. The direct involvement of cuproptosis, however, still needs to be demonstrated and the results should be biochemically and genetically validated.

Despite the above, cuproptosis is a distinct form of copper-dependent cell death that is mechanistically separate from apoptosis, ferroptosis, necroptosis and from nonspecific oxidative injury [[50], [51], [52]]. The mechanism of cuproptosis is closely linked with mitochondrial respiration and selectively preys on lipoylated enzymes of the TCA cycle. Whilst the transporter SLC31A1 enables uptake of cellular copper [53]. SLC25A3 delivers copper from the mitochondrial inner membrane and helps to maintain mitochondrial copper homeostasis and mitochondrial protein copper-loading, in cytochrome-c oxidase biogenesis [54,55]. Under conditions of pathological copper overload or copper-ionophore-induced copper delivery, extra amounts of copper are extruded into mitochondria [56]. The reductase, FDX1, carries out copper reduction and regulates mitochondrial protein lipoylation [57]. Copper then binds lipoylated TCA-cycle proteins (in particular DLAT), causing abnormal protein oligomerization, destabilization of iron–sulfur-cluster proteins, mitochondrial proteotoxic stress and cell death [58]. Therefore, increased SLC31A1 expression, Cu accumulation, altered FDX1 or LIAS expression and oxidative stress should be seen as cuproptosis-related changes but not necessarily as evidence for cuproptosis in the absence of those downstream biochemical and functional events. Because these defining downstream events were not directly examined here, our findings support SLC31A1-related Cu dyshomeostasis, but they do not establish cuproptosis as the mode of hepatocyte death.

There is increasing evidence that oxidative stress and inflammatory response contribute to the development of insulin resistance and diabetes [59]. Inflammation is a risk factor for oxidative stress, and oxidative stress at elevated levels cause decreased insulin sensitivity by impairing insulin signaling and glucose uptake in the liver cells [60]. In this study, we found that the hepatocyte-specific knockdown of SLC31A1 decreased pro-inflammatory cytokines at serum and mRNA levels, and also, this knockdown increased the influx of macrophages and neutrophils were decreased. Moreover, in the hepatocyte-specific SLC31A1 knockdown model, antioxidant enzyme activity was restored through the Nrf2 pathway. Accumulation of copper is known to cause oxidative stress and inflammation, which are both primary risk factors for the development of T2D-related hepatic dysfunction [61]. Knockdown of SLC31A1 activates Nrf2, which is a master factor for antioxidant response, and thus reducing oxidative damage and inflammation. These effects are crucial for breaking the vicious cycle of metabolic dysfunction, because oxidative stress increases insulin resistance and lipotoxicity.

PI3K/Akt signaling pathway was also considered to be related to glucose production and glycogen synthesis under diabetes conditions [62]. Besides, AMPK is an important regulator of glucose and fatty acid metabolism by controlling glucose uptake, glycogen synthesis and fatty acid oxidation in the liver, mainly involving in diabetes [49,[63], [64], [65]]. In this work, we found that knockdown of SLC31A1 can activate AMPK, but not PI3K/Akt. Knockdown of AMPK could reverse the metabolic benefits of SLC31A1 knockdown in T2D mice. These results showed that the beneficial effects of SLC31A1 knockdown are dependent on AMPK, suggesting the therapeutic potential of activating AMPK to attenuate diabetes-induced liver disorders. These findings connect hepatocytes SLC31A1, copper metabolic modification, AMPK and hepatic metabolic disease in diabetes, and show that targeting SLC31A1 or copper homeostasis could have therapeutic effects in the liver disease associated with T2D. Provided hepatocyte-specific SLC31A1 knockdown increased AMPK signaling, whereas AMPK inhibition could only partially abolish its improvement in glucose and lipid metabolism, suggesting that other mechanisms might also be involved. Lack of evidence for upstream regulator of AMPK, copper-dependent mitochondrial bioenergetics or a direct interaction between SLC31A1 and AMPK are added as limitations. Studies will be needed to determine the molecular link between SLC31A1-dependent accumulation of copper and regulation of AMPKα1.

Pharmacological doses of Dorsomorphin reversed some metabolic benefits of hepatocyte-specific SLC31A1 knockdown in T2D mice. Yet, because Dorsomorphin has known AMPK-independent activities and a T2D + control-shRNA + Dorsomorphin group was not present, these animal data should be considered to be largely correlative, rather than definitive evidence of involvement of the AMPK pathway. In cultured hepatocytes, SLC31A1 knockdown selectively recovered the level of AMPKα1 phosphorylation under glucosamine- or oleic acid-induced metabolic stress. Furthermore, genetic depletion of AMPKα1, but not of AMPKα2, reverted the effects of SLC31A1 knockdown on glucose production and TG accumulation. All of these findings supported AMPKα1 as a downstream mediator of SLC31A1-dependent regulation of hepatocyte metabolism.

## 5 Conclusions

In conclusion, hepatocyte SLC31A1 upregulation is associated with hepatic Cu accumulation and metabolic dysfunction in T2D mice. SLC31A1 knockdown improves glucose and lipid metabolic abnormality, oxidative stress, inflammation and liver injury. Thus, our findings suggest that the SLC31A1-copper homeostasis axis might be a potential target of a therapeutic pathway for diabetes-associated hepatic metabolic dysfunction. Cellular genetic experiments identify AMPKα1, but not AMPKα2, as the candidate downstream factor of the effects of SLC31A1 knockdown for glucose production and TG production. However, liver-specific genetic experiments will precede the AMPKα1 dependency *in vivo*. Further study is needed to determine whether cuproptosis is directly involved in this pathogenesis and to unravel the SLC31A1-dependent Cu accumulation-mediated mechanism to regulate AMPK. In addition, SLC31A1 functional evidence is mainly based on loss-of-function experiments *in vivo*. No SLC31A1 gain-of-function experiments were performed. Future rescue and overexpression studies are needed to elucidate whether increased or restoring expression of SLC31A1 had an effect on copper homeostasis, AMPKα1 activation and glucose and lipid metabolism in T2D mice.

## 6 Limitations

Several limitations deserve comment in our study. The human relevance of our current results remains speculative. Indeed, analysis of the Human Protein Atlas and Tabula Muris databases confirms the predominant expression of SLC31A1 in hepatocytes, but these databases do not show the pathophysiological role of SLC31A1 in human diabetic liver disease. No liver samples from people with T2D were examined. Nor were patient-level liver or blood copper concentrations and associations of SLC31A1 expression with metabolic phenotypes recorded in these patients. Further validation with human diabetic liver tissues, clinically well-defined patient collections and physiologically relevant human hepatic tissues or models are needed to study the clinical importance of the SLC31A1–copper homeostasis axis. Despite increased expression of SLC31A1, accumulation of hepatic copper and changed expression of FDX1, LIAS, DLAT and DLD implying disruption of copper homeostasis and involvement of a cuproptosis-related mechanism, these observations alone do not prove that cuproptosis is the involved type of hepatocyte death. Direct evidence such as aggregation of lipoylated DLAT, loss of iron–sulfur-cluster proteins, failure of mitochondria respiration, rescue by chelation of copper and genetic inhibition of FDX1, LIAS and protein lipoylation was not recorded in the current study. More, TUNEL staining reveals DNA fragmentation, but can only distinguish among apoptosis, cuproptosis and other types of cell damage. Thus, our present findings should be regarded as pointing out that there exists SLC31A1-dependent copper dyshomeostasis and hepatocellular injury but no proof of cuproptosis. Future investigation using biochemical, genetic and mitochondrial functional studies will be required to understand whether cuproptosis may directly contribute to diabetic liver dysfunction. Dorsomorphin/Compound C is not a selective AMPK inhibitor and can affect BMP type-I receptors and other AMPK-independent pathways. Besides, animal study did not have a T2D + control-shRNA + Dorsomorphin group. So, the direct metabolic and hepatic actions of chronic Dorsomorphin treatment cannot be disentangled from apparent suppression of the SLC31A1-knockdown phenotype and the pharmacological experiments do not provide clear evidence of an *in vivo* interaction between SLC31A1 and AMPK. Although the cellular genetic experiments showed that AMPKα1 depletion, but not AMPKα2 depletion, negated the effects of SLC31A1 knockdown on both glucose production and triglyceride accumulation, neither were hepatocyte-specific AMPKα1 depletion experiments done; thus, the present data support a role of AMPKα1 for cultured hepatocytes, but do not strongly distinguish an AMPKα1 dependence *in vivo*. Future studies of AMPKα1-deficient mice with hepatocyte-specific knock-out, together with an appropriate balanced factorial design, are needed to validate the relationship of SLC31A1 and AMPKα1 in diabetic liver dysfunction. HepG2 cells are a transformed hepatoma cell line and have metabolic and mitochondrial features distinct from normal hepatocytes. We, for example, did not examine insulin-stimulated Akt signaling and the ability of insulin to inhibit glucose production in insulin-stimulated cells. We also did not examine mitochondrial respiratory dependence and copper-dependent cell death, so the cellular experiments did not set up cuproptosis. We did not reproduce our observations with primary mouse hepatocytes, primary human hepatocytes or another non-transformed human hepatic model. Thus, the cellular conclusions were limited to the HepG2 models and will need future studies in primary and physiologically relevant hepatocyte systems to assess the translational relevance of the observed SLC31A1-dependent effects. Finally, results obtained only in male mice must be confirmed in female mice and also in studies explicitly designed to assess sex-by-treatment effects.

## CRediT authorship contribution statement

**Yin-Qin Cheng:** Writing – review & editing, Writing – original draft, Project administration, Methodology, Investigation, Formal analysis, Data curation, Conceptualization. **Min Wei:** Resources, Investigation, Formal analysis, Data curation, Conceptualization. **Le Yang:** Validation, Methodology, Investigation, Formal analysis, Data curation. **You-Yi Zhuang:** Visualization, Validation, Software, Resources, Methodology, Investigation, Formal analysis. **Qing-Bo Lu:** Writing – review & editing, Writing – original draft, Supervision, Data curation, Conceptualization.

## Ethics declaration

This study was conducted in accordance with the ARRIVE (Animal Research: Reporting of In Vivo Experiments) guidelines. This study was approved by the Laboratory Animal Ethics Committee of Jiangnan University. (Approval No. JN.NO20230130d034013[101]).

## Declaration of generative AI and AI-assisted technologies in scientific writing process

In the preparation of this manuscript, we use a generative artificial intelligence-assisted tool Kimi alone for language formatting and readability polishing. After using such tools, all the authors have critically reviewed and revised the draft, and all the authors share full and equal responsibility for the published final content.

## Financial support

This work was supported by the Clinical Research and Translational Medicine Research Program of Affiliated Hospital of 10.13039/501100004028Jiangnan University (LCYJ202306), the General Research Project of Nantong Health Commission (MS2025045), the 10.13039/501100001809National Natural Science Foundation of China (82500439), Natural Science Foundation of Jiangsu, China (BK20250297), Wuxi Science and 10.13039/100006180Technology Development Fund Project “Light of the Taihu Lake” (K20221028), Wuxi Municipal 10.13039/100018696Health Commission Youth Project (Q202226), 10.13039/100018696Top Talents Supporting Plan for young and middle-aged people of Wuxi Health Committee (HB2023045).

## Declaration of competing interests

The authors declare that they have no known competing financial interests or personal relationships that could have appeared to influence the work reported in this paper.

## Data Availability

Data will be made available on request.
