# Supplementary material for: Hepatocyte SLC31A1-dependent copper accumulation contributes to hepatic glucose and lipid metabolic disturbances through AMPKα1 inactivation
Source: Biochem Biophys Rep. 2026 Sep 11;48:102787. doi: 10.1016/j.bbrep.2026.102787 (PMC13584062; doi:10.1016/j.bbrep.2026.102787)
Supplement: Supplementary file 1 [file mmc1.pdf]

Supplementary data

A

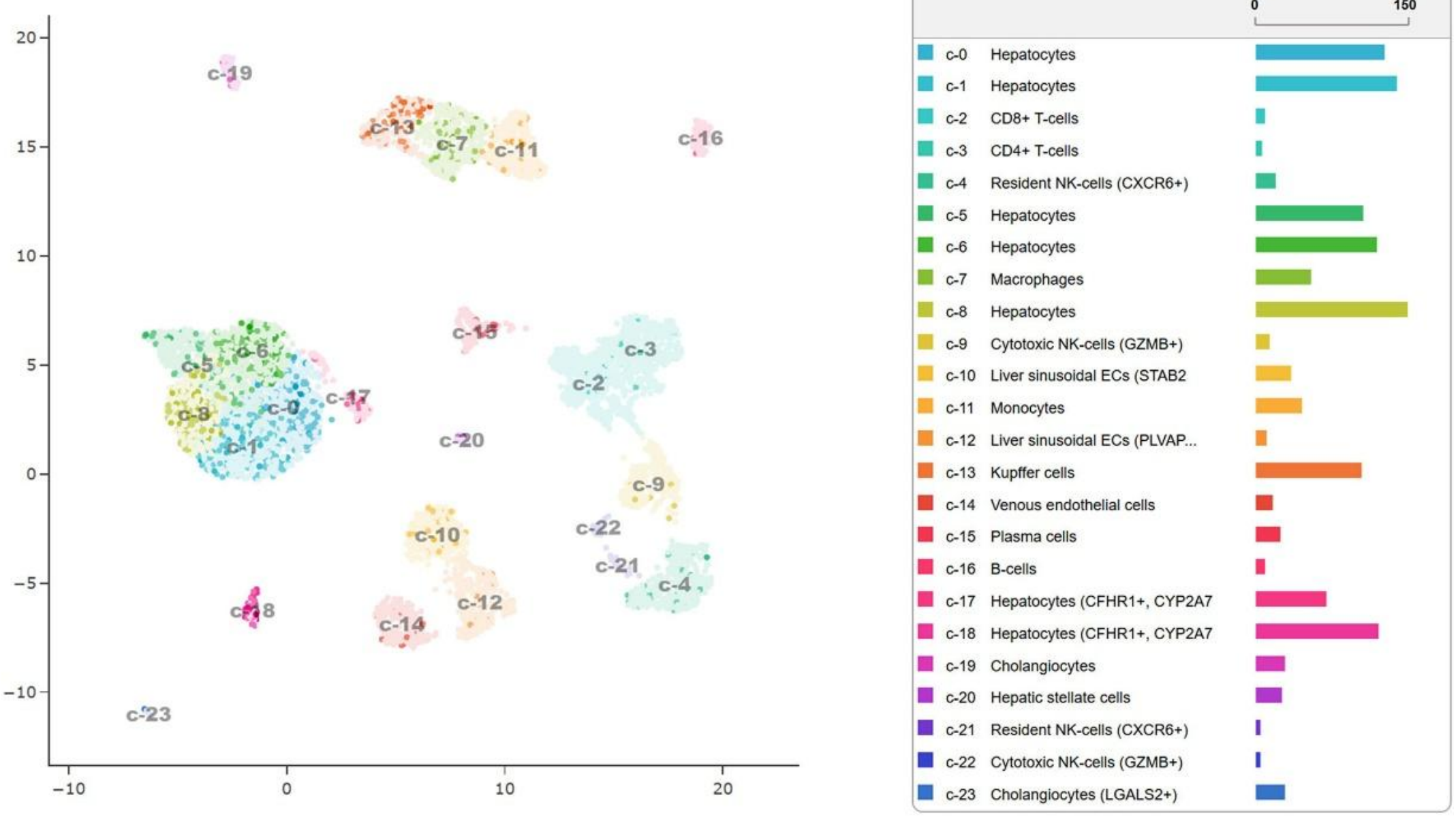

B

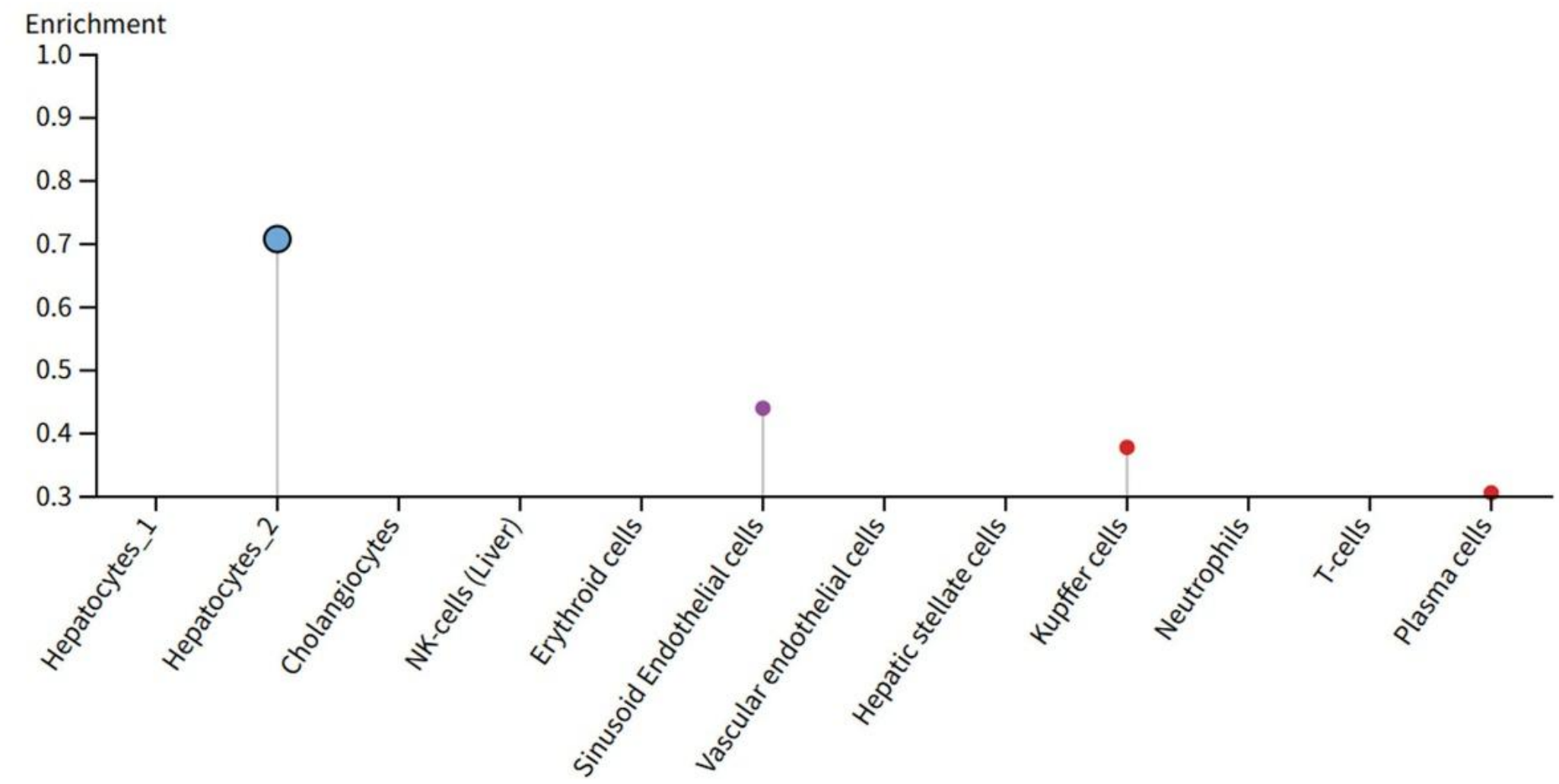

Figure S1. The online database of HUMAN PROTEIN ATLAS shows the expression of SLC31A1 in different cell types with the liver system (A,B).

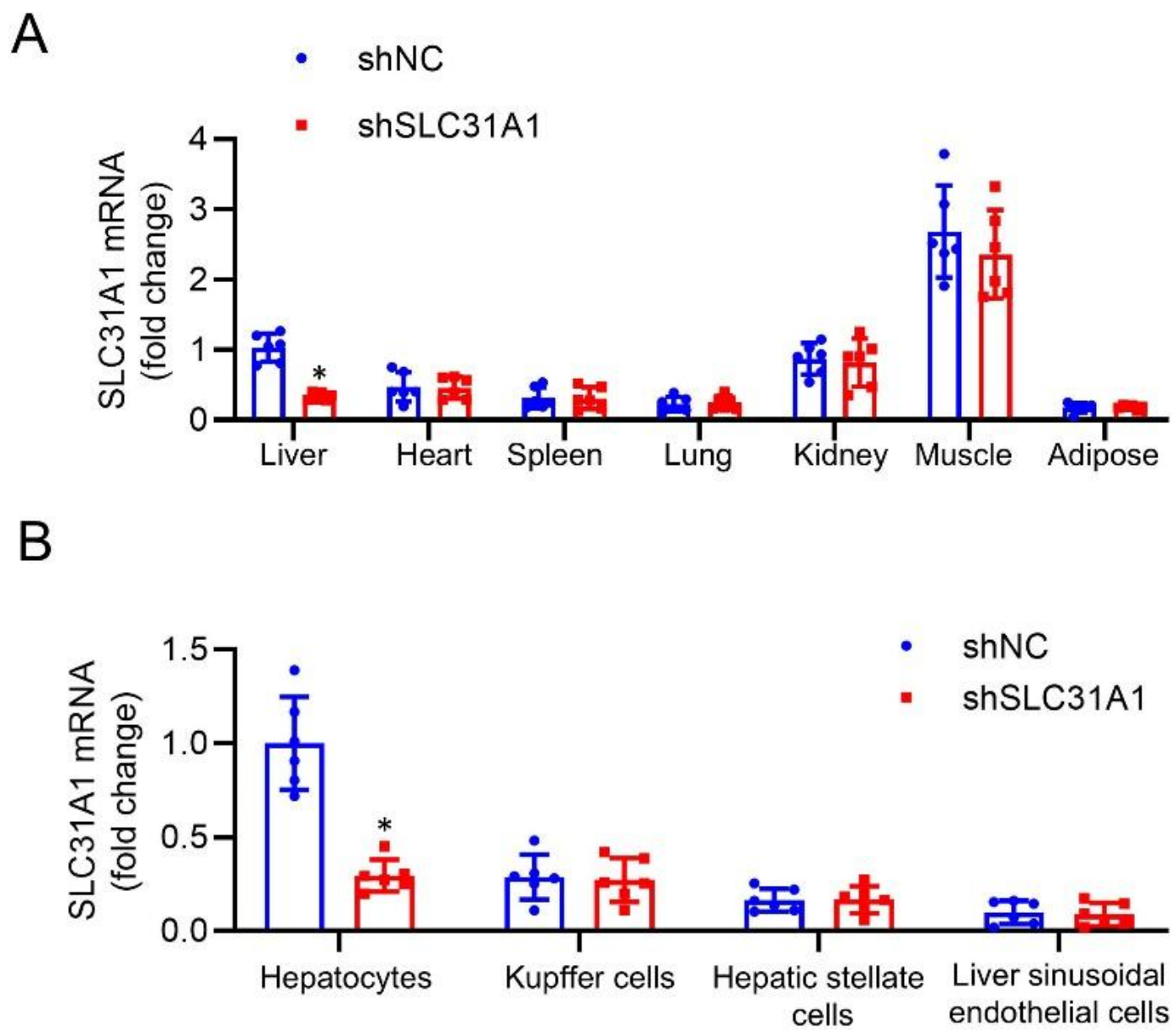

Figure S2. The expression of SLC31A1. (**A**) The mRNA level of SLC31A1 in the liver, heart, spleen, lung, kidney, gastrocnemius muscle and inguinal adipose tissue after SLC31A1 knockdown. (**B**) The mRNA level of SLC31A1 in isolated primary hepatocytes, Kupffer cells, hepatic stellate cells, and liver sinusoidal endothelial cells. n=6 for each group. For comparisons between two groups, an unpaired two-tailed Student's *t* test was used. *$P < 0.05$ *vs*. shNC.

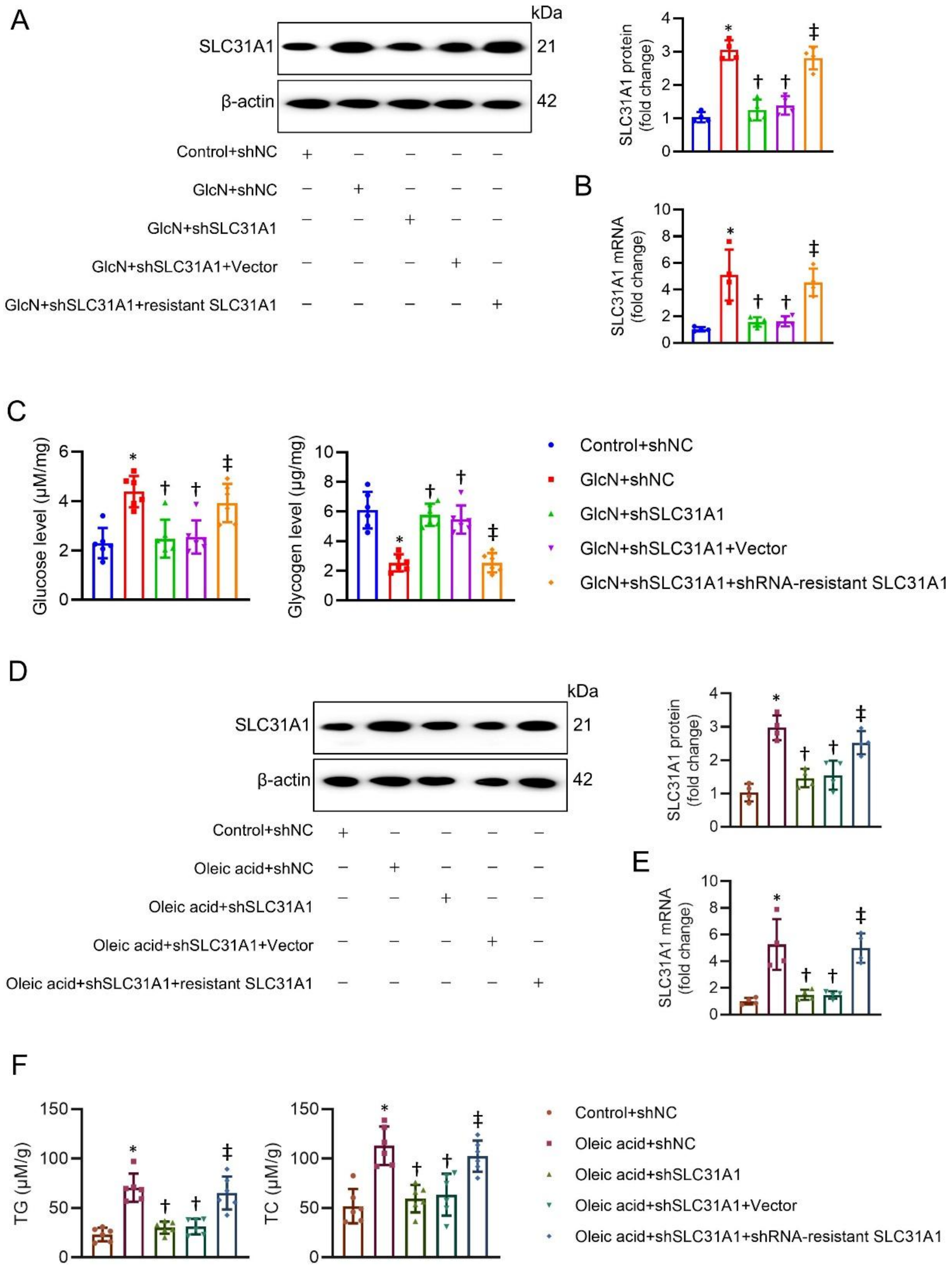

Figure S3. On-target rescue experiments confirm the specificity of SLC31A1 knockdown effects. (**A**) Western blot analysis of SLC31A1 protein expression in hepatocytes treated with control shRNA (shNC) or SLC31A1 shRNA (shSLC31A1), followed by re-expression of shRNA-resistant SLC31A1 (shSLC31A1 + Rescue) or empty vector, under GlcN-treated conditions. (**B**) Relative SLC31A1 mRNA levels in the same groups as in (A), measured by qRT-PCR. (**C**) Glucose production (left) and

glycogen content (right) in GlcN-treated hepatocytes from each group. (**D**) Western blot analysis of SLC31A1 protein expression in oleic acid-stimulated hepatocytes under the same knockdown and rescue conditions. (**E**) Relative SLC31A1 mRNA levels in OA-stimulated cells corresponding to (D). (**F**) The contents of TG and TC in OA-stimulated hepatocytes from each group. n=4-6 for each group. For comparisons among multiple groups, one-way ANOVA followed by Bonferroni-adjusted post-hoc comparisons was used. *$P<0.05$ *vs*. Control+shNC, †$P<0.05$ *vs*. GlcN+shNC or Oleic acid+shNC, ‡$P<0.05$ *vs*. GlcN+shSLC31A1+Vector or Oleic acid+shSLC31A1+Vector.

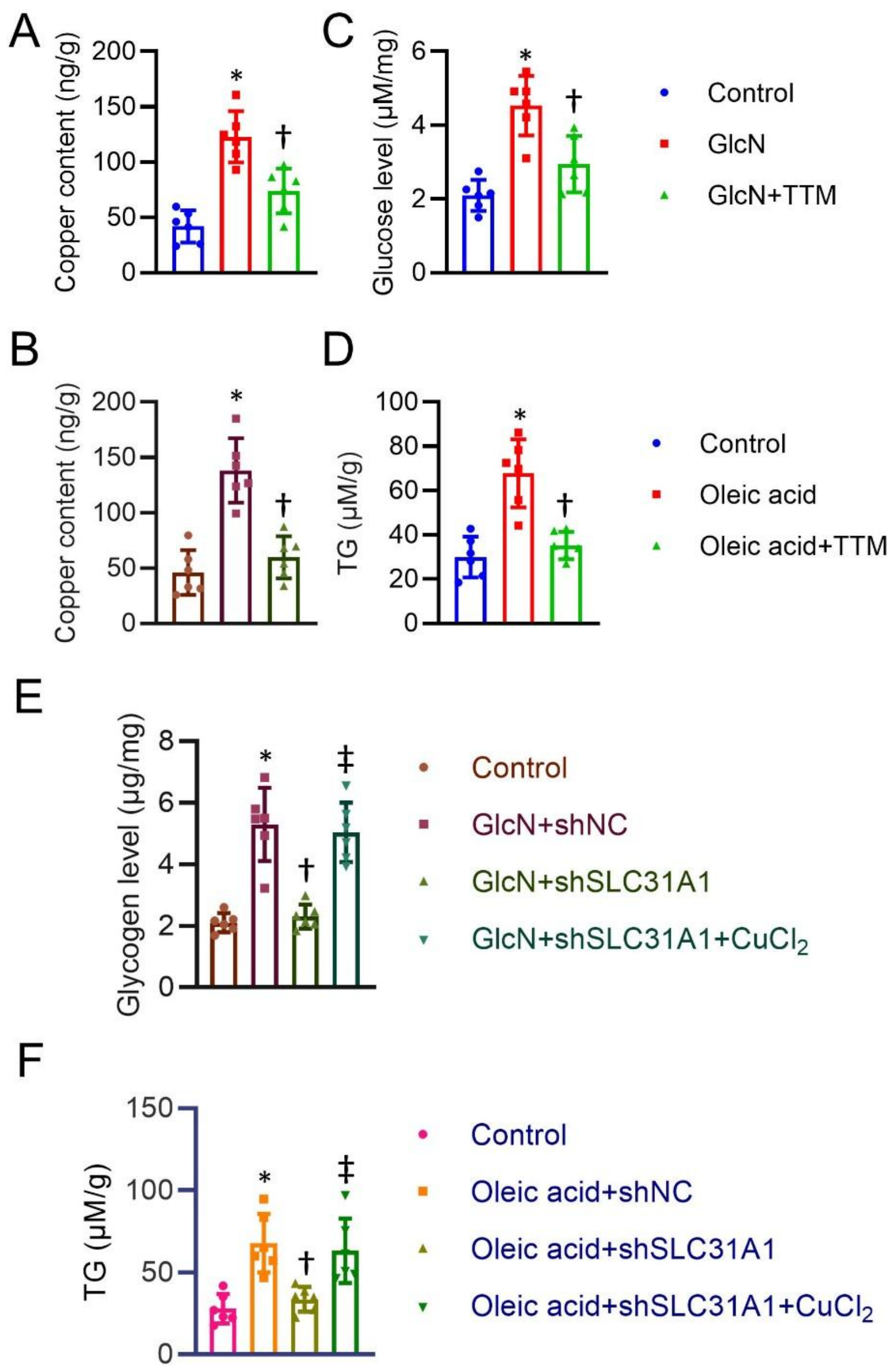

Figure S4. TTM treatment mimicked the reduction in glucose production and TG accumulation observed with SLC31A1 knockdown, whereas $CuCl_2$ supplementation partially reversed these phenotypes. (**A**) Intracellular copper levels in hepatocytes treated with vehicle or the copper chelator tetrathiomolybdate (TTM, 10 μM). (**B**) Effects of TTM treatment on glucose production in GlcN-treated cells. (**C**) Intracellular copper levels in hepatocytes treated with vehicle or the copper chelator tetrathiomolybdate (TTM, 10 μM). (**D**) Effects of TTM treatment on glucose production in Oleic acid-treated cells. (**E-F**) Hepatocytes with SLC31A1 knockdown were treated with or without $CuCl_2$ to assess the levels of glucose and triglyceride (TG). n=4-6 for each group. For comparisons among multiple groups, one-way

ANOVA followed by Bonferroni-adjusted post-hoc comparisons was used. *$P < 0.05$ *vs*. Control, †$P < 0.05$ *vs*. GlcN, Oleic acid, GlcN+shNC or Oleic acid+shNC, ‡$P < 0.05$ *vs*. GlcN+shSLC31A1 or Oleic acid+shSLC31A1.

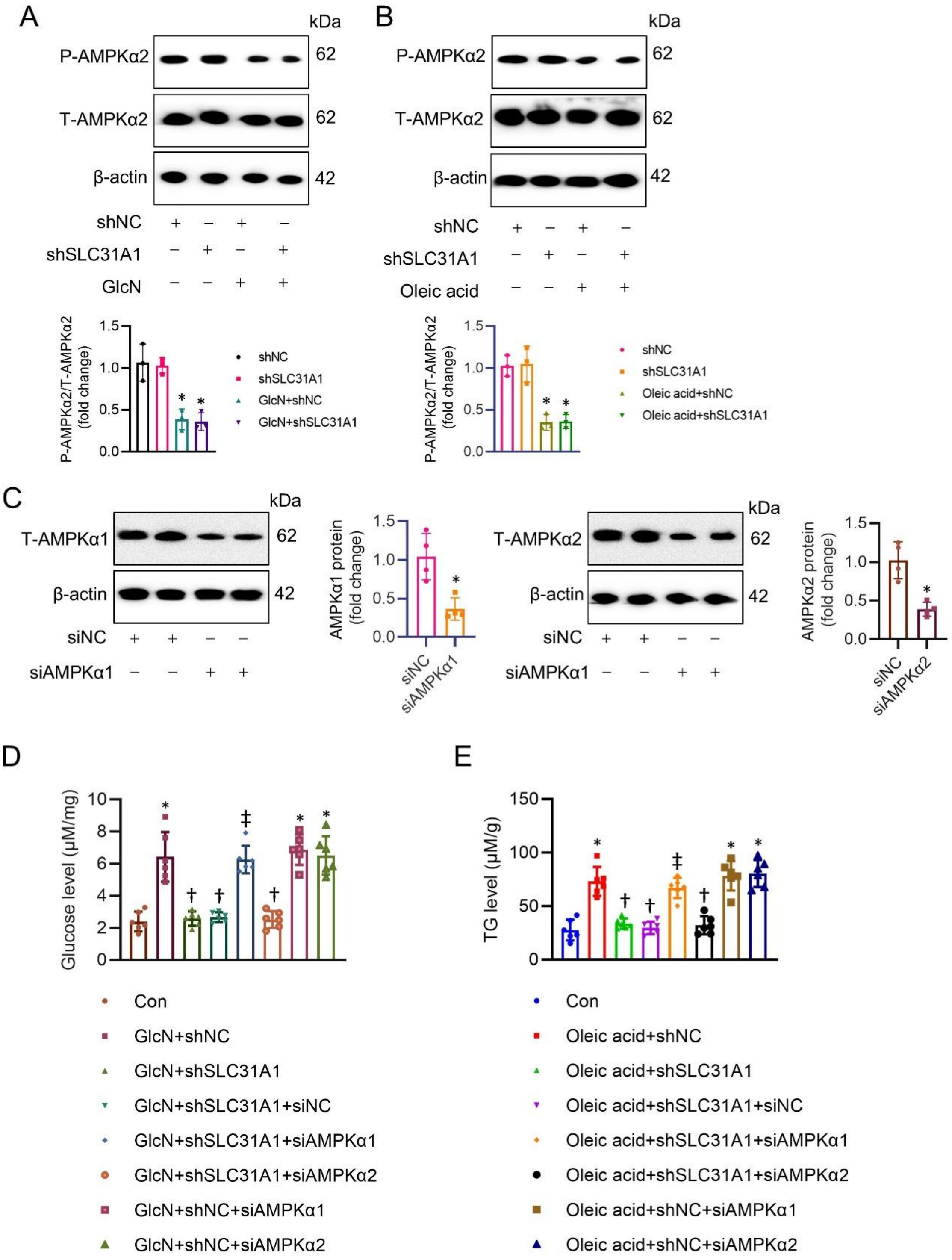

Figure S5. AMPKα1, but not AMPKα2, mediates the metabolic effects of SLC31A1 knockdown. (**A-B**) Representative Western blots and quantification of phospho-AMPKα2 (p-AMPKα2) and total AMPKα2 protein levels in hepatocytes treated with GlcN or Oleic acid in the presence or absence of SLC31A1 knockdown. (**C**) Validation of siRNA-mediated knockdown of AMPKα1 and AMPKα2 in hepatocytes. (**D-E**) Effects of individual depletion of AMPKα1 or AMPKα2 on

glucose production and intracellular triglyceride (TG) accumulation in SLC31A1-knockdown hepatocytes under GlcN or OA stimulation. n=5-6 for each group. For comparisons among multiple groups, two-way ANOVA followed by Tukey's HSD post-hoc comparisons was used (A-B). For comparisons between two groups, an unpaired two-tailed Student's *t* test was used (C). For comparisons among multiple groups, one-way ANOVA followed by Bonferroni-adjusted post-hoc comparisons was used (D-E). *$P<0.05$ *vs*. shNC, siNC, or Control (Con), †$P<0.05$ *vs*. GlcN, Oleic acid, GlcN+shNC or Oleic acid+shNC, ‡$P < 0.05$ *vs*. GlcN+shSLC31A1+siNC or Oleic acid+shSLC31A1+siNC.

Table S1. The information for RT-PCR in mice.

Gene	Primer sequences (5’-3’)
LIAS	Forward: GTCCCGGACAAGAGTACATGG
	Reverse: TGTGTCCCCCATCAACAT GA
SLC31A1	Forward: TATGAACCACACGGACGACAA
	Reverse: GCCATTTCTCCAGGTGTATTGA
ATP7B	Forward: ACCTATCAGCCTTACCTCATTCA
	Reverse: GTGCCCCAGGGTCTCAAAAT
MTF1	Forward: GCGGAAGTGACGCTAGGGA
	Reverse: GAAAGGTAACGGCCTTTCTCCG
LIPT1	Forward: AACTGCGTCTAGAGGGCTGA
	Reverse: AAGTGGAATTAGCATGCTGCC
GLS	Forward: TTCGCCCTCGGAGATCCTAC
	Reverse: CCAAGCTAGGTAACAGACCCT
CDKN2A	Forward: CGAACTCGAGGAGAGCCATC
	Reverse: TACGTGAACGTTGCCCATCA
PDHA1	Forward: TCATCACTGCCTATCGAGCAC
	Reverse: GTTGCCTCCATAGAAGTTCTTGG
PDHB	Forward: CTGTGTTGTCATTCGGCAGC
	Reverse: TCCAGCAAAGCCCATCTCTG
DLD	Forward: GAGCTGGAGTCGTGTGTACC
	Reverse: TCACGTCAGCCTCAATTGGTT
DLAT	Forward: GGCACAAGGGATGTTCCTCTG
	Reverse: TGTGGCTTTAAGCTGGTCACT
FDX1	Forward: TAACAGTCCACTTCAAGAACCGA
	Reverse: CACAACATCTAGCAGAGAGTCG
PEPCK	Forward: CTGCATAACGGTCTGGACTTC
	Reverse: GCCTTCCACGAACTTCCTCAC
G6pase	Forward: AAAAAGCCAACGTATGGATTCCG

	Reverse: CAGCAAGGTAGATCCGGGA
FASN	Forward: GTTGGCCCAGAACTCCTGTA
	Reverse: GTCGTCTGCCTCCAGAGC
Srebp1	Forward: TGGTTGTTGATGAGCTGGAG
	Reverse: GGCTCTGGAACAGACACTGG
Dgat1	Forward: TCACCACACACCAATTCAGG
	Reverse: GACGGCTACTGGGATCTGA
Scd1	Forward: CACCTGCCTCTTCGGGATTT
	Reverse: TCTGAGAACTTGTGGTGGGC
PPARα	Forward: CAGTGGGGAGAGAGGACAGA
	Reverse: AGTTCGGGAACAAGACGTT
Cpt-1	Forward: AGTGGCCTCACAGACTCCAG
	Reverse: GCCCATGTTGTACAGCTTCC
Hmgcs2	Forward: ATACCACCAACGCCTGTTATG
	Reverse: CAATGTCACCACAGACCACCA
IL-1β	Forward: AAATGCCACCTTTTGACAGTG
	Reverse: GGATGCTCTCATCAGGACAG
IL-6	Forward: AGCAGCATCACCTTCGCTTAG
	Reverse: TGTCCAGATATTGGCATGGG
TNF-α	Forward: CTGAACTTCGGGGTGATCGG
	Reverse: GGCTTGTCACTCGAATTTTGAGA
GAPDH	Forward: GGTTGTCTCCTGCGACTTCA
	Reverse: GGTGGTCCAGGGTTTCTTACTC

Table S2 Primer sequences for PCR analysis in hepatocytes.

Gene	Sequence
FASN	5'-AGTACACACCCAAGGCCAAG-3' (Forward)
	5'–GTGGATGATGCTGATGATGG-3' (Reverse)
Srebp1	5'-GCCCAGGTGACTCAGCTATT-3' (Forward)
	5'-GCATCCGAGAATTCCTTGTC-3' (Reverse)
Dgat1	5'-GGCATCCTGAACTGGTGTGTG-3' (Forward)
	5'-GAGCTTGAGGAAGAGGATGGTG-3' (Reverse)
Scd1	5' -TGGGTTGGCTGCTTGTG-3' (Forward)
	5' -GCGTGGGCAGGATGAAG-3' (Reverse)
PPARα	5' -CCAGTATTTAGGAAGCTGTCCTG-3' (Forward)
	5' -CGTTGTGTGACATCCCGACAG-3' (Reverse)
Cpt-1	5' -TGAGCGACTGGTGGGAGGAG-3' (Forward)
	5' -GAGCCAGACCTTGAAGTAGCG-3' (Reverse)
Hmgcs2	5' -GGAACCCATATGGAGAATGTGT-3'(Forward)
	5' -ATCGCTGCCAGCTTGCTT-3' (Reverse)
PCPEK	GCAAGACGGTTATCGTCACCC
	GGCATTGAACGCTTTCTCAAAAT
G6pase	GTGTCCGTGATCGCAGACC
	GACGAGGTTGAGCCAGTCTC
GAPDH	5'-CACTGCCACCCAGAAGA-3' (Forward)
	5'-GCTTCCCGTTCAGCTCA-3' (Reverse)
